# An improved assembly and annotation of the melon (*Cucumis melo* L.) reference genome

**DOI:** 10.1038/s41598-018-26416-2

**Published:** 2018-05-24

**Authors:** Valentino Ruggieri, Konstantinos G. Alexiou, Jordi Morata, Jason Argyris, Marta Pujol, Ryoichi Yano, Satoko Nonaka, Hiroshi Ezura, David Latrasse, Adnane Boualem, Moussa Benhamed, Abdelhafid Bendahmane, Riccardo Aiese Cigliano, Walter Sanseverino, Pere Puigdomènech, Josep M. Casacuberta, Jordi Garcia-Mas

**Affiliations:** 1 Centre for Research in Agricultural Genomics (CRAG) CSIC-IRTA-UAB-UB, Campus UAB, Barcelona, 08193 Spain; 2 IRTA (Institut de Recerca i Tecnologia Agroalimentàries), Barcelona, 08193 Spain; 3 Faculty of Life and Environmental Sciences, University of Tsukuba, Tsukuba, 305-8572 Japan; 4 Institute of Plant Sciences Paris-Saclay (IPS2), INRA, CNRS, University of Paris-Sud, University of Evry, University Paris-Diderot, Sorbone Paris-Cite, University of Paris-Saclay, Orsay, 91192 France; 5 Sequentia Biotech SL, Barcelona, 08193 Spain

## Abstract

We report an improved assembly (v3.6.1) of the melon (*Cucumis melo* L.) genome and a new genome annotation (v4.0). The optical mapping approach allowed correcting the order and the orientation of 21 previous scaffolds and permitted to correctly define the gap-size extension along the 12 pseudomolecules. A new comprehensive annotation was also built in order to update the previous annotation v3.5.1, released more than six years ago. Using an integrative annotation pipeline, based on exhaustive RNA-Seq collections and *ad-hoc* transposable element annotation, we identified 29,980 protein-coding loci. Compared to the previous version, the v4.0 annotation improved gene models in terms of completeness of gene structure, UTR regions definition, intron-exon junctions and reduction of fragmented genes. More than 8,000 new genes were identified, one third of them being well supported by RNA-Seq data. To make all the new resources easily exploitable and completely available for the scientific community, a redesigned Melonomics genomic platform was released at http://melonomics.net. The resources produced in this work considerably increase the reliability of the melon genome assembly and resolution of the gene models paving the way for further studies in melon and related species.

## Introduction

In 2009, the first plant whole genome shotgun sequence was released using exclusively next-generation sequencing technologies^1^. Since then, many reference plant genomes have been completed^2^. The availability of the genome sequences of many model plants and crops has provided new opportunities for identifying the genes underlying important traits, for studying genome evolution and for helping in the process of plant breeding. However, the plant genome sequences reported until now differ in the quality of their assemblies, in many instances representing good quality drafts still far from being complete. For this reason, improving reference genome assemblies is an important objective in many plant species. There are several examples of plant *de novo* genome assemblies that have been substantially improved using optical mapping^3,4^. Optical mapping consists in the production of DNA fingerprints that are used to construct the map of the genome. Optical maps offer a relatively straight-forward and independent assessment of any genome assembly that claims to provide chromosome-level contiguity. The automation of this technology has been commercially available through the BioNano Genomics Iris or the OpGen Argus systems^5,6^. The availability of single-molecule real-time sequencing technologies and high-resolution optical mapping is now allowing further improving the quality of complex reference genomes^7^. Updating a genome’s annotation over time is a challenging and complex task^8^. Building new annotations upon the foundation of existing annotations appears as a necessary step to ensure continuity, in particular for reference genomes, providing incremental means to move forward in light of new data.

The melon (*Cucumis melo* L.) genome sequence was assembled and annotated in 2012^9^. A genomic platform was also built in order to host the genome sequence (available at http://melonomics.net). The published genome assembly of the double-haploid line DHL92 (v3.5) consisted of 375 Mbp containing 1,594 scaffolds and 29,865 contigs, with a N50 scaffold size of 4.68 Mbp with 90% of the assembly contained in 78 scaffolds^9^. The transposable element (TE) fraction was annotated, accounting for 19.7% of the genome assembly. A total of 27,427 protein-coding genes and 34,848 transcripts were annotated in this first version. The melon genome was organized in 12 pseudomolecules, with 87.5% of the scaffold assembly anchored to a genetic map. Following the release of the first draft genome v3.5, the melon genome was subsequently revised to improve the anchoring and orientation of the scaffold assembly using a targeted SNP selection strategy, obtaining the v3.5.1 assembly^10^ (available at http://melonomics.net). In this new genome version, 98.2% and 90% of the scaffold assembly was anchored and oriented to a SNP genetic map, respectively, representing a substantial improvement of the pseudomolecules. 27.8 Mb of scaffold assembly remained unanchored in pseudomolecule 0 and the genome annotation used in the melon genome release v3.5.1 was the same as in v3.5. Additionally, the reference genome line DHL92 was re-sequenced and by combining the Illumina paired-end reads with the Post-Assembly Genome-Improvement Toolkit (PAGIT), which is a tool to obtain annotated genomes from contigs, allowed a substantially reduction of the number of small contigs and Ns in the genome assembly^11^.

Taking advantage of the continuous improvement of the genomics technologies and bioinformatics approaches^4,7^ here we describe an improved version of the melon genome assembly (v3.6.1), which includes an optical mapping correction and re-orientation of some scaffolds along the 12 pseudomolecules. Moreover, the availability of additional melon RNA-Seq data collections^12–16^ as well as the possibility to rely on a specific TE annotation^17^ and more efficient pipelines^18^ allowed for a new annotation (v4.0) accounting for 29,980 protein-coding genes. The new genome annotation, hosted in a redesigned Melonomics platform (http://melonomics.net), offers refined gene structures, new gene models and improved functional descriptions.

## Results and Discussion

### An optical mapping approach allowed improving pseudomolecules

To improve the melon genome assembly v3.5.1, an optical mapping approach^19–21^ was undertaken in collaboration with the company OpGen.

OpGen collected 21 high density MapCards from all Argus-generated MapCards with the *Nco*I enzyme (see Materials and Methods), to obtain single molecule restriction maps (SMRMs) for the melon genome. We obtained 350,646 DNA molecules with a total size of 146.46 Gbp. The average size of the molecules was 417.69 Kbp and the corresponding average size of the *Nco*I-digested fragments was 11.95 Kbp. An in silico *Nco*I-digested profile of the v3.5.1 assembly and SMRMs were later used as input data for the Genome-Builder^TM^ assembler (OpGen Inc.), which generated a new assembly with 27 superscaffolds (hereafter called “optical assembly”; Supplementary Table S1).

**Figure 1:**
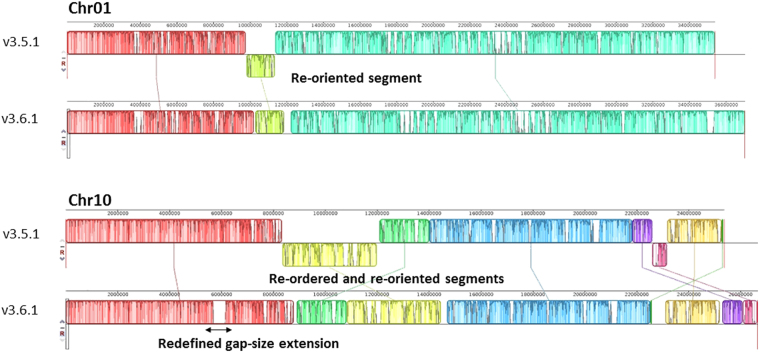
Examples of OpGen correction. The chromosomes are laid out horizontally and homologous segments between v3.5.1 and v3.6.1 are shown as colored blocks. Each color block stands for a scaffold or consecutive merged scaffolds. Blocks that are shifted downwards represent re-oriented segments in v3.6.1. Blocks that are swiped/translocated in another position represent re-ordered segments. Gap size correction is shown as white gaps inside the blocks.

In total, 105 out of the 141 scaffolds of the v3.5.1 assembly (74%) were used for generating the superscaffolds, reaching a total length of 315.5 Mbp. For scaffolds with size larger than 200 Kbp, N50 and N90 values for the optical assembly were 15.52 Mbp and 6.9 Mbp, respectively, 3.4x and 4.3x higher than the respective N50 and N90 values of the v3.5.1 assembly (N50: 4.57 Mbp, N90: 1.61 Mbp). Additionally, optical mapping determined the orientation of 16 scaffolds, previously annotated as “non-oriented” in the v3.5.1 assembly, and corrected the orientation of 5 scaffolds. Alignment of in silico *Nco*I-digested scaffold profiles of the v3.5.1 assembly versus the consensus optical maps allowed for the establishment of the real gap size between neighbouring scaffolds within the same superscaffold. The actual size of 74 gaps was estimated to be 25.5 Mbp, with a maximum gap of 1.52 Mbp, a minimum of 813 bp and an average gap size of 344 Kbp. Combined analyses of the OpGen-generated superscaffolds and the anchoring data of the v3.5.1 assembly led to the generation of the v3.6.1 assembly. Table 1 and Supplementary Table S2 present a comparison between assemblies v3.5.1 and v3.6.1 in terms of chromosome and N-regions (gaps) length. The new assembly (excluding chromosome 0), containing 143 scaffolds, spans a total size of 375.3 Mbp, corresponding to a size increase of 5.74% with respect to the v3.5.1 assembly. The major part of this increase was due to the determination of actual gap sizes (a 5.21% increase). A synteny block representation, highlighting the changes/corrections between the two assemblies v3.5.1 and v3.6.1 is reported in Fig. 1 and Supplementary Figure S1.

**Table 1:** Comparison between v3.5.1 and v3.6.1 assemblies in terms of chromosome length and gaps extension (number of Ns).

Chromosome	v3.5.1		v3.6.1	
	length (bp)	number of Ns (bp)	length (bp)	number of Ns (bp)
Chr01	35,383,099	4,224,098	37,037,532	5,746,973
Chr02	26,193,771	2,467,907	27,064,691	3,272,777
Chr03	29,387,469	3,090,555	31,666,927	5,286,237
Chr04	33,123,230	3,484,828	34,318,044	4,557,078
Chr05	28,337,775	3,556,980	29,324,171	4,428,209
Chr06	35,939,859	4,056,829	38,297,372	6,313,150
Chr07	26,773,857	2,703,377	28,958,359	4,821,595
Chr08	32,513,408	3,874,538	34,765,488	5,785,898
Chr09	24,107,567	2,533,680	25,243,276	3,572,934
Chr10	25,362,315	2,705,038	26,663,822	3,957,926
Chr11	31,442,130	3,210,864	34,457,057	5,604,798
Chr12	26,400,393	2,792,021	27,563,660	3,864,732
**Total**	**354,964,873**	**38,700,715**	**375,360,399**	**57,212,307**

### Transposon and repeat elements annotation

Taking advantage of the progress on transposable element (TE) annotation tools, we performed a new and more comprehensive TE annotation resulting in an increase on TE coverage from 19.7%^9^ to up to 44%. Class I transposons are the most abundant TEs and account for 33.2% of the genome (14.7% in the previously published annotation). DNA transposons coverage also increased up to 7.9% of the genome. The remaining TE space corresponds to unclassified elements. This comprehensive TE annotation is of particular interest for interspecific comparisons of the TE content and distribution as it has been performed using REPET^17^, a pipeline that is now widely used for TE annotation in plants. In addition, this annotation will also be very useful for studies on TE dynamics and their impact on the genome. However, as a fraction of the TE-related sequences are small and can be found intermingled with genes, a comprehensive annotation may in some cases make gene annotation challenging. Therefore, an additional reduced TE annotation was generated for masking the genome and facilitate gene annotation (see Material and Methods). This reduced TE annotation covers 34% of the genome.

### Structural and functional genome annotation

Continuous refinement and routine updates of annotation are prerequisites for correctly interpreting the functional elements of a genome. An integrated approach was used in this work to update the v3.5.1 melon protein-coding genes^9,10^. The structural annotation workflow relied on the MAKER v2 software^18^, which uses a combination of tools to integrate evidence from transcriptome assembly and *ab initio* gene discovery. The previous annotation release (v3.5.1) and an automated-produced annotation provided by NCBI (v102, https://www.ncbi.nlm.nih.gov/) were transferred to the new genome assembly v3.6.1 through a liftover process. The transferred annotations were used also as EST tracks to train the definition of HMM models for the *ab initio* gene discovery step. The integrated annotation workflow **(**Supplementary Figure S2**)** uses RNA-Seq data to add additional features to existing gene models and to identify new gene models where none existed previously. Next-generation sequencing data, especially RNA sequencing data, have already been reported to hold great potential for an independent confirmation and improvement of genome annotation offering an unprecedented improvement for genome annotation analysis^22–27^. Among the several genomic resources available for melon a large RNA-Seq collection exists that includes public^12–16^ and private experiments. A subgroup of 57 RNA-Seq datasets **(**Supplementary Table S3**)** coming from 20 different tissues and developmental stages derived from four melon genotypes was chosen to obtain a reliable genome-guided transcriptome assembly. About 2 billion reads were collected and after the trimming and filtering processes, about 70% of them were retained, representing 540-fold coverage of the v3.6.1 melon genome. Benchmarking analysis were conducted in order to test if a different number of reads might be associated with an increasing benefit in terms of gene annotation. Although the use of only 10% of the total reads was sufficient to produce more than 50% of the maximum number of transcripts detected, the use of larger datasets constantly improved the transcriptome definition. A similar trend was also reported in the maize genome annotation^8^. Therefore, the complete set accounting for 1.2 billion reads (about 220 billion bp) was used for the final melon genome-guided transcriptome assembly. 105,000 assembled transcripts were predicted by TRINITY with an average length of the coding sequence (CDS) of about 1 Kbp and all of them were used to support the gene annotation process.

The integration of the transcriptome assembly, the evidence derived from the liftover of the previous annotations and the HMM models coming from the *ab initio* gene discovery allowed the annotation of 43,564 protein-coding genes. Among these genes, 13,584 were masked since they overlapped with repeat elements, leaving 29,980 protein-coding genes into a final comprehensive v4.0 melon annotation. The complete list of masked genes is reported in Supplementary Table S4. As expected, most of the masked genes showed sequence similarity with sequences potentially coding for transposases, retrotransposon proteins, Gag-pro-like proteins as well as with unknown genes.

In order to achieve a qualitative description of the functional annotation, the final set of 29,980 protein-coding genes was annotated using an Automatic assignment of Human Readable Descriptions (AHRD)^28^. AHRD annotation uses a combination of approaches and databases to assign a coherent description and a reliability score to each annotation overcoming problems caused by wrong annotations, lack of similar sequences and partial alignments. These advantages can produce relevant insight when exploring a gene annotation. Other plant and non-plant genomes have been annotated using a similar approach, including the 6a maize annotation^8^ and tomato ITAG3.2 (https://www.solgenomics.net/). Among the 29,980 genes, the AHDR procedure allowed to assign a function description to 24,247 of them. About 72% of the AHRD annotated genes have a three star AHRD score and 22% a two star score. Only 6% showed a zero star score. A gene ontology annotation and a KEGG enzymatic pathways association with the annotated genes is reported in Supplementary Table S5.

### Evaluation of the quality of the annotation

**Figure 2:**
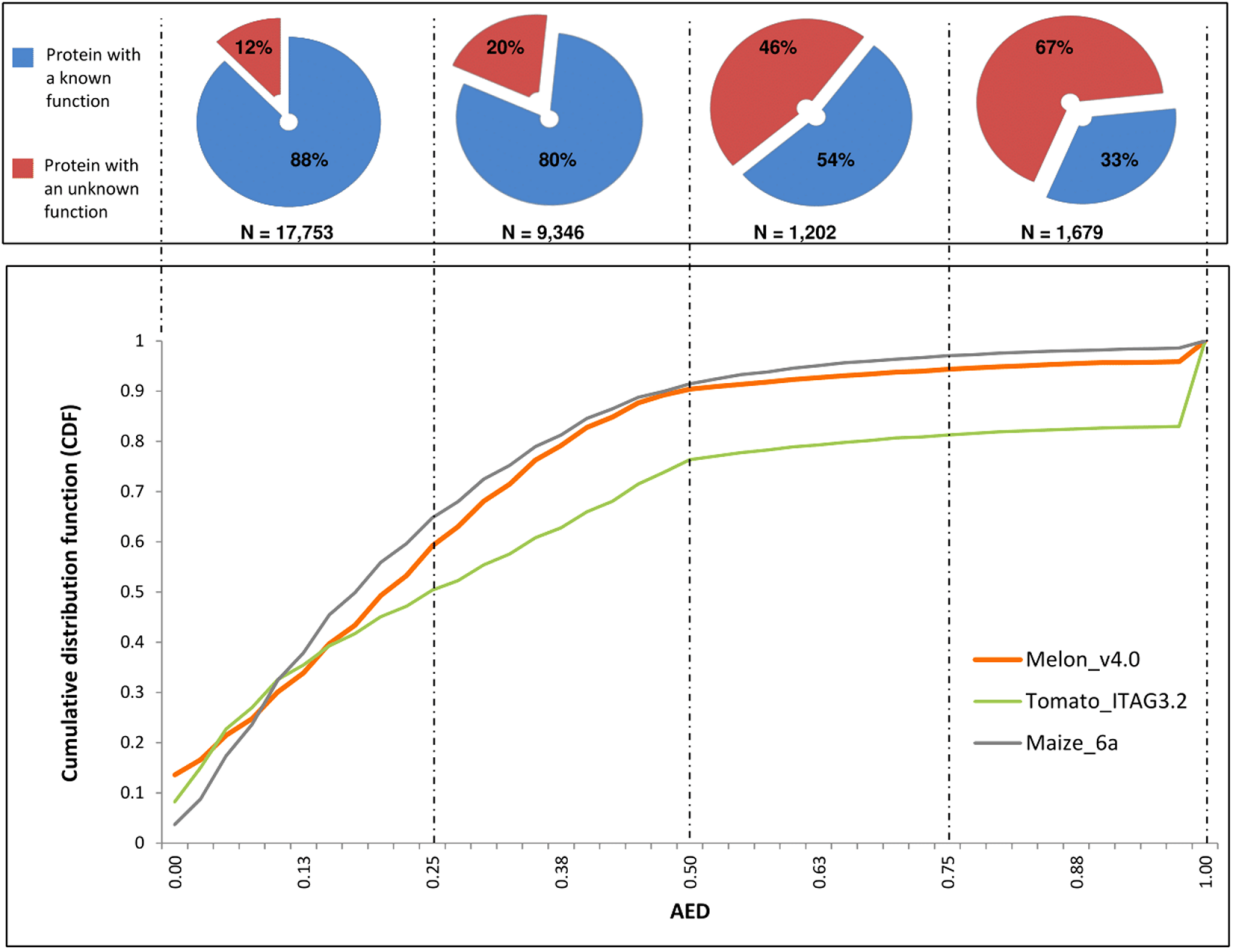
AED analyses of the the Melon v4.0 gene models (orange line) in comparison with the Tomato ITAG3.2 (green line) and Maize 6a (grey line) annotations. Shown on the y axis is the cumulative distribution of AED for each dataset. In the upper part of the graph is shown the ratio of the proteins with a known function versus proteins with an unknown function for each quartile of the AED range.

A quantitative evaluation of the accuracy of the exon-intron structure is a critical step towards obtaining a gold standard annotation^29^. MAKER v2 uses a performance measure called annotation edit distance (AED) to assess the accuracy of the genome annotation^18^. AED measures the goodness of fit of an annotation to the evidence supporting it. AED is a number between 0 and 1, with 0 denoting perfect concordance with the available evidences and 1 indicating a lack /absence of support for the annotated gene model. Figure 2 shows the cumulative distribution function (CDF) of AED for the v4.0 melon annotation in comparison with the most recent release of tomato (ITAG3.20) and maize (6a) annotations, which were obtained by using a similar strategy. More than 90% of the v4.0 annotations have an AED score of less than 0.5 with a profile of CDF close to the maize 6a annotation and similar to that of the tomato ITAG3.2 annotation.

Figure 2 also shows the distribution of annotated genes with a known function *versus* genes with an unknown function for each quartile of the CDF. An increasing percentage of genes with an unknown function is observed moving towards the third and fourth quartiles. This pattern reflects the decreasing quality of the annotations presenting AED scores greater than 0.5. It is also plausible that these genes encode small peptides, pseudogenes or non-coding RNAs. By contrast, it is noteworthy that about 1,000 of the 4,000 genes with an unknown function of the first and second quartiles are supported by RNA-Seq and could represent orphan genes, which are reported to have important roles in plant and non-plant organisms^30^.

**Figure 3:**
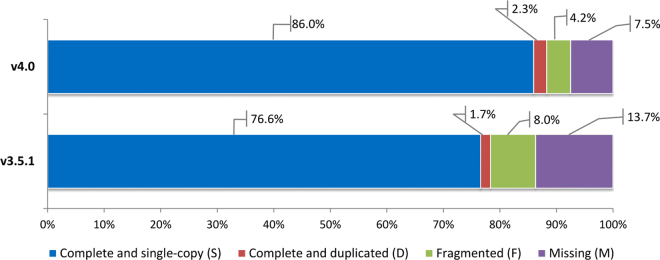
Busco completeness comparison between v3.5.1 and v4.0 melon annotations based on Embriophyta odb9 dataset. Colors in the bar represent the different classes of the Busco assessment results.

To evaluate the improvements of the v4.0 release in comparison with the previous v3.5.1 release, we compared the two annotations to a core set of plant genes using BUSCO^31^. The core set includes near-universal single-copy orthologue genes selected from OrthoDB v9, which were used to assess the completeness and the goodness of gene content. The results revealed that about 88.3% of the genes were complete, and only less than 7.5% and 4.2% were missing or fragmented, respectively (Fig. 3). When compared with results of the previous melon annotation v3.5.1, an improvement of about 10% was reached with complete genes. The improvements are mainly related to the reduction of fragmented genes (from 8% to 4.2%) and to the recovering of missing genes (from 13.7% to 7.5%). Although the BUSCO assessment for plant genomes relies on a reduced set of 1,440 genes, the observed trend corroborates the expected enhancements due to an improved genome assembly and to a more reliable annotation based on RNA-Seq evidences.

A gene model comparison between the v4.0 and the previous annotation v3.5.1 resulted in the identification of 8,350 new genes and in the deletion of about 6,000 v3.5.1 genes. An overlap for about 21,000 loci was also observed, even though only for 7,500 of them a perfect gene model correspondence across the two releases was found. By contrast, a redefinition of the gene structure was made up for about 13,500 genes. In most of the cases it only concerned the addition, deletion or modification of UTR regions as well as the collapsing of the alternative transcripts from the v3.5.1 version. A relevant improvement concerned the merging of 5,400 split genes of the v3.5.1 into 2,400 of the v4.0. Examples of improved gene annotations in v4.0 are shown in Supplementary Figure 3, among them the virus resistance gene VPS41. In the previous version v3.5.1 the VPS41 gene was split in two consecutive genes MELO3C004831T1 and MELO3C004827T1, annotated as Auxin-induced protein 22D and Beta-amyrin synthase, respectively. In the new release v4.0 the two genes have been fused into one gene, MELO3C004827.2, annotated as a vacuolar protein sorting-associated protein 41 homolog^32^.

The annotation approach used in this work resulted in the definition of 8,350 novel protein-coding loci. About 40% of these (3,060) were supported by the RNA-Seq data. Interestingly, we observed that most of these novel genes showed low expression levels, which may explain why they escaped prior annotations. The newly discovered genes could give new insight in melon research and could fill gaps in the identification of candidate genes underlying important traits. Finally, about 6,000 v3.5.1 genes were not present in the new annotation v4.0. All of them, although successfully predicted in the annotation workflow and present in the raw dataset of 43,564 genes, were removed since there was overlapping with repeat elements. We also observed that these removed genes were frequently short (<100 bp), monoexonic or without a known functional description. By contrast, only a small part (<10%) showed a description or an expression signal in at least one tissue, evincing a possible role as functional genes. The main differences between the v3.5.1 and v4.0 annotation releases, concerning different feature parameters, such as gene length and number of exons, are reported in Supplementary Table S6.

### Melonomics v2, a new genomic platform in melon

**Figure 4:**
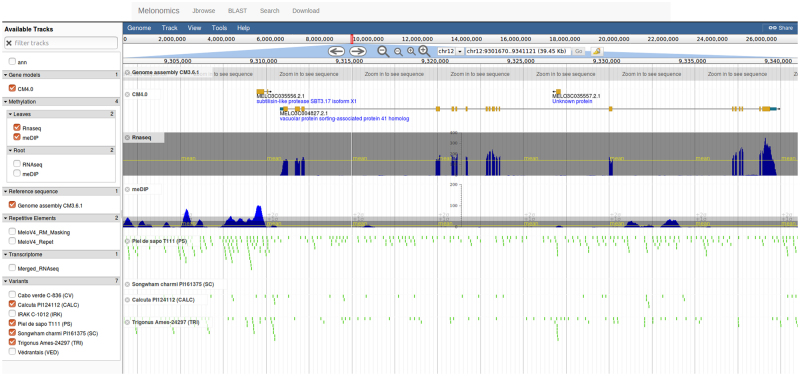
The JBrowse navigator included in the new designed Melonomics.net web page. The browser integrates gene structures and various kinds of evidences at the genomic, transcriptomic and epigenomic level, including the v4.0 gene models, two TE annotations (MeloV4_Repet and MeloV4_RM_Masking), a cumulative RNA-Seq expression track (Merged RNA-Seq), epigenetic marks derived from methylation (MeDIP) of leaf and root tissues jointly with the corresponding RNA-Seq tracks and a variome set including the re-sequencing of seven melon accessions.

To make all the new resources easily exploitable and completely available for the scientific community, a newly designed Melonomics genomic platform has been released at http://melonomics.net. In addition to the previous Melonomics version that is still active and accessible from the main hub page, the new Melonomics webpage offers new tools and functionalities. In particular, it hosts a customized instance of JBrowse^33^. The browser integrates gene structures and various kinds of evidences at the genomic, transcriptomic and epigenomic level. Below the reference genome sequence axis, the browser incorporates 15 tracks including the v4.0 gene models, two TE annotations (MeloV4_Repet and MeloV4_RM_Masking), a cumulative RNA-Seq expression track, epigenetic marks derived from methylation of leaf and root tissues jointly with the corresponding RNA-Seq tracks and a variome set including the re-sequencing of seven melon accessions (Fig. 4). The tracks can be browsed via the hierarchical track selector. The browser also offers bulk download of interval-specific track data in common file formats (FASTA, GFF3, BED) as well as a search tool (elasticsearch) that allows performing searches by key words directly from the browser. The versatility of the Jbrowse also permits to integrate user-provided datasets, either local or hosted on third party servers. While searching or scrolling, the page URL is dynamically updated with sufficient information to fully reproduce the display. This feature enables collaboration through URL sharing between remote users. The integration of all the different information permits easy highlighting of the behaviour of a specific feature or of an entire chromosome.

Besides the Jbrowse, the Melonomics webpage also hosts a Search section, where users can check a gene or transcript based on keywords, ID, position and pathway, among others. Gene conversion between the previous and the new genome release can be identified in order to track gene updates between versions, and sequences can be searched with Blast using the previous and the new melon genome assemblies and annotations. Finally, a Download section allows the users to retrieve all the data and resources related to the new melon genome assembly and annotation.

## Material and Methods

### DNA extraction for Optical mapping

High molecular-weight (HMW) DNA was extracted from the double-haploid line DHL92. Fifteen g of young leaves were harvested after a pre-treatment of 48 h in the dark. Intact nuclei were prepared and embedded in agarose plugs as described by Zhang *et al*.^34^. Nuclei lysis was performed after incubation in the lysis buffer (0.5 M EDTA, pH 9.0–9.3, 1% sodium lauryl sarcosine, 0.1 mg/mL proteinase K) for 48 h at 45 °C. Plugs containing HMW DNA were washed once in 0.5 M EDTA, pH 9.0–9.3 for 1 h at 50 °C, once in 0.05 M EDTA, pH 8.0 for 1 h on ice, and stored in 0.05 M EDTA, pH 8.0 at 4 °C. Optical mapping was performed by OpGen, Inc. Optimal restriction enzyme for optical mapping for the v3.5.1 assembly was selected by OpGen after testing 13 different restriction enzymes and evaluating different parameters of the restriction profile, such as average fragment size, total size of usable sequence, size and number of large fragments and presence of repeat fragments. Overall evaluation showed that *Nco*I was the most appropriate enzyme for optical-based genome assembly (Supplementary Table S7). Individual molecules of HMW DNA were elongated and fixed onto Optical Chips (MapCards)^35^, digested with *Nco*I and stained with fluorescent dyes. Restriction fragments were imaged by laser-illuminated fluorescent microscopy using the Argus Mapper.

Bulk data collection of MapCards were obtained and super-scaffolding was performed using Genome-Builder^TM^ software. Superscaffolds were used to map them manually onto the v3.5.1 assembly, in order to determine the level of scaffold anchoring concordance between the two assemblies. Mapchart^36^ and MUAVE (http://darlinglab.org/mauve/mauve.html) were used for genome graphical representations and for synteny analysis between v3.5.1 and v3.6.1.

### Repeats annotation

Total transposable element (TE) annotation was performed with the REPET package^17^, with default parameters and thresholds. Annotations shorter than 200 bp were discarded.

TEs can contain complete or partial genes, which includes coding domains, and this can lead to leave unannotated coding genes if TE representatives contain those domains. For this reason, confusing TE representatives due to sequence quality or TE representatives that contained specific non-TE protein domains were removed from the masking for the gene annotation process. In order to do so, we built a dedicated subset of the REPET annotation. TE representatives obtained in the TEdenovo step of REPET pipeline were checked with hmmscan (HMMER 3.1b1, hmmer.org) against the PFAM database^37^ for coding domains. TE representatives with known domains of non-TE proteins (kinases, NB-ARC, LRR); with Ns content higher than 30% and/or TE representatives defined as “noCat” were discarded. For the remaining TE representatives, TE copies search was performed with repeatMasker (http://repeatmasker.org) with a cutoff of 250. Again, TE annotations shorter than 200 bp were discarded. Final nucleotide coverage of this partial annotation was of 34%.

### Structural and functional annotation

#### Transcriptome assembly

We assessed 57 RNA-Seq datasets for the Transcriptome assembly. In an effort to minimize false gene models discovery, only paired-end, strand-specific Illumina HiSeq reads were used. This ensured a considerable reduction of miss-mapped reads and increased the accuracy of the transcriptome assembly. About 100 additional RNA-Seq melon datasets were also available as January 2017 in NCBI SRA archive but were not considered since most of them (>90) derived from a RIL collection and/or from single reads layout experiments. The complete list of datasets used with a detailed sample description is reported in Supplementary Table S3.

All RNA-Seq reads were processed through a quality check and trimming pipeline using FastQC (http://www.bioinformatics.babraham.ac.uk/projects/fastqc) and Trimmomatic^38^ respectively, to remove residual adapters and low-quality sequences. Sequences from each library were aligned to the v3.6.1 genome using STAR^39^. The parameteres used are reported in Supplementary Table S8. Then, samtools^40^ was used to filter only proper-paired reads and optical duplicates were removed using picard-tools (v1.110).

Libraries were checked to have a proportion of mapped reads > 70%. We used raw counts generated by featureCounts (v1.4.6)^41^ to evaluate gene expression level across the datasets.

The transcriptome was then assembled using TRINITY (version 2.3.2)^42^ using the genome-guided mode (see Supplementary Table S8 for the parameters used) and all the mapped reads. The redundancy of the assembly was then reduced by using CD-HIT-EST (version 4.6.6) (see Supplementary Table S8 for the parameters used). The quality of the final assembly was evaluated with BUSCO (version 3)^31^ using the conserved plant genes as database (OrthoDB v9). The general statistics of the assembly were produced with TransRate (version 1.0.3)^43^.

#### Structural annotation with Maker v2

For a complete structural annotation of the melon genes, the Maker v2^18^ pipeline was used. All the following steps were performed using the Maker v2 configuration file, only the parameters different from the default values are indicated. First, a repeat masking step was performed using the Eukaryotic database with Repeat Masker. A lift-over of the melon transcripts v3.5.1 and the melon NCBI transcripts (ASM31304v1) were then performed (see Supplementary Table S8 for the parameters used). The obtained GFF3 were filtered to retain only the transcripts with an AED < = 0.3 and then they were used to create an *ab initio* HMM matrix with SNAP. The final annotation was produced by using: 1) the GFF3s obtained after the lift over of the v3.5.1 and the NCBI transcripts, 2) the genome guided assembled FASTA of the transcripts and 3) the SNAP HMM model (see Supplementary Table S8 for the parameters used).

#### Functional annotation

The functional annotation of the genes was performed with the AHRD pipeline (v3.3.3) (https://github.com/groupschoof/AHRD). The predicted protein sequences obtained after the structural annotation step were BLASTed against the TrEMBL database of melon protein sequences (downloaded on April 2017, DB1), the UniRef database of Viridiplantae (downloaded on April 2017, DB2) and the TrEMBL database of Viridiplantae (downloaded on April 2017, DB3). The parameters of the blastp were the following: -qcov_hsp_perc 15 -evalue 0.001. The obtained tables were used as input for the AHRD pipeline assigning a weight of 854, 904 and 653 to the DB1, DB2 and DB3, respectively. AHRD assigns a quality-code that consists of a three-character string (“*” if the respective criteria is met or “−” if not) to each annotation based on three parameters: i) the overlap of the blast results among the databases used, ii) the overcoming of threshold for e-value (<1E-10) and bit score (>50) and iii) the evaluation of the top token score of assigned Human-Readable-Description (HRD). Finally, the KEGG enzymatic pathways and GO terms were associated with the predicted proteins by using Interproscan (version 5.19-58.0) with the following parameters: -appl PANTHER, Pfam, SUPERFAMILY, Coils, SMART, CDD, PIRSF -pa -goterms.

An effort was made in this work to preserve a close link with the previous annotations, in terms of gene names. For genes overlapping between v3.5.1 and v4.0 we maintained the same ID than in the previous release (e.g. MELO3C000001) except for the addition of “.2” to highlight that this is a second release (e.g. MELO3C000001.2). For fused genes we used the ID of the larger gene. For the new gene models predicted a new ID was assigned. A conversion table between v3.5.1 and v4.0 releases is given in Supplementary Table S5.

### Methylated-DNA Immunoprecipitation, MeDIP-seq sequencing and transcriptomic analysis

Genomic DNA was isolated from roots and leaves of the cultivar Charentais (*Cucumis melo* L. subsp. *melo* var *cantalupensis*) using E.Z.N.A Plant DNA Kit (Omega). Fragmentation was performed using Diagenode Bioruptor 200 UCD-300 (30 s then off 90 s for 25 cycles, low power position). The following steps were performed using Diagenode Auto hMeDIP Kit in the SX-8G IP-Star® Compact System. Anti-5-methylcytosine antibody (NA8133D3, Merck Millipore, Diagenode) was used for precipitation. DNA was then purified using Auto Ipure kit v2 (Diagenode). Libraries were synthetized using NebNext Ultra DNA Library Preparation Kit (NEB) according to the manufacturer’s instructions and sequenced by Illumina technology. The same melon accession was used to collect the the total RNA from roots of 10 day old (cultivated *in vitro*) and from leaves of 3 weeks old, grown in a growth chamber (long day conditions, temperature: 27 °C (day) and 21 °C (night), relative humidity: 60%). The Nucleospin RNA kit (Macherey-Nagel) was used for the extraction according to the manufacturer’s instructions. Libraries were synthetized from 2 µg of total RNA using NEBNext Ultra Directional RNA library Preparation Kit (NEB) according to the manufacturer’s instructions. Two biological replicates were analysed for each tissue. Libraries were sequenced by Illumina technology.

### Melonomics. Genomic platform development

Data relative to GO terms, KEGG pathways, gene and transcript names, its coordinates and descriptions was imported into a MySQL (version 5.5) based relational database stored in a CentOS server (version 7.4). This database was then integrated into a graphical interface by using AngularJS and SocketIO technologies in the front-end and NodeJS (version 6.12) together with the Express framework (version 4.15) in the back-end. The site also contains a BLAST section, in which the user can query any sequence against different versions of the melon genome, transcriptome, and proteome with blat (version 36x2), blastn, blastp and blastx (version 2.6). A JBrowse (version 1.12.3) was also integrated to the site, thus allowing visual inspection of the new melon assembly with its corresponding gene annotation, the variants found in different melon ecotypes and other omics data (methylation levels and RNA-Seq expression).

### Data availability statement

The melon genome assembly v3.6.1 and annotation v4.0 are available at http://melonomics.net.

## Electronic supplementary material

Supplementary information

Supplementary Table S4

Supplementary Table S5
