# Supplementary material for: An improved assembly and annotation of the melon (Cucumis melo L.) reference genome
Source: Sci Rep. 2018 May 24;8:8088. doi: 10.1038/s41598-018-26416-2 (PMC5967340; doi:10.1038/s41598-018-26416-2)
Supplement: Supplementary file 1 — Supplementary information [file 41598_2018_26416_MOESM1_ESM.pdf]

Supplementary information

An improved assembly and annotation of the melon (*Cucumis melo* L.) reference genome

Valentino Ruggieri[1,2], Konstantinos G. Alexiou[1,2], Jordi Morata[1], Jason Argyris[1], Marta Pujol[1,2], Ryoichi Yano[3], Satoko Nonaka[3], Hiroshi Ezura[3], David Latrasse[4], Adnane Boualem[4], Moussa Benhamed[4], Abdelhafid Bendahmane[4], Riccardo Aiese Cigliano[5], Walter Sanseverino[5], Pere Puigdomènech[1], Josep M Casacuberta[1], Jordi Garcia-Mas[1,2*]

[1]Centre for Research in Agricultural Genomics (CRAG) CSIC-IRTA-UAB-UB, Campus UAB, Bellaterra, Barcelona, Spain

[2]IRTA (Institut de Recerca i Tecnologia Agroalimentàries), Barcelona, Spain

[3]Faculty of Life and Environmental Sciences, University of Tsukuba, Tsukuba, 305-8572, Japan

[4]Institute of Plant Sciences Paris-Saclay (IPS2), INRA, CNRS, University of Paris-Sud, University of Evry, University Paris-Diderot, Sorbone Paris-Cite, University of Paris-Saclay, Orsay, France

[5]Sequentia Biotech SL, 08193 Bellaterra, Barcelona (Spain)

Supplementary Figures

Supplementary Figure S1. Optical mapping correction for the 12 chromosomes of the v3.6.1 assembly. Syntenic blocks (consisting of one or more consecutive scaffolds) between v3.5.1 and v3.6.1 are connected by a line. Re-oriented blocks are shifted downwards on the v3.5.1 while the reordered blocks are shown as translocated blocks in the v3.6.1. Gaps are shown as white spaces in the blocks. Histograms inside each box represent the level of similarity between homologous parts of the two assemblies.

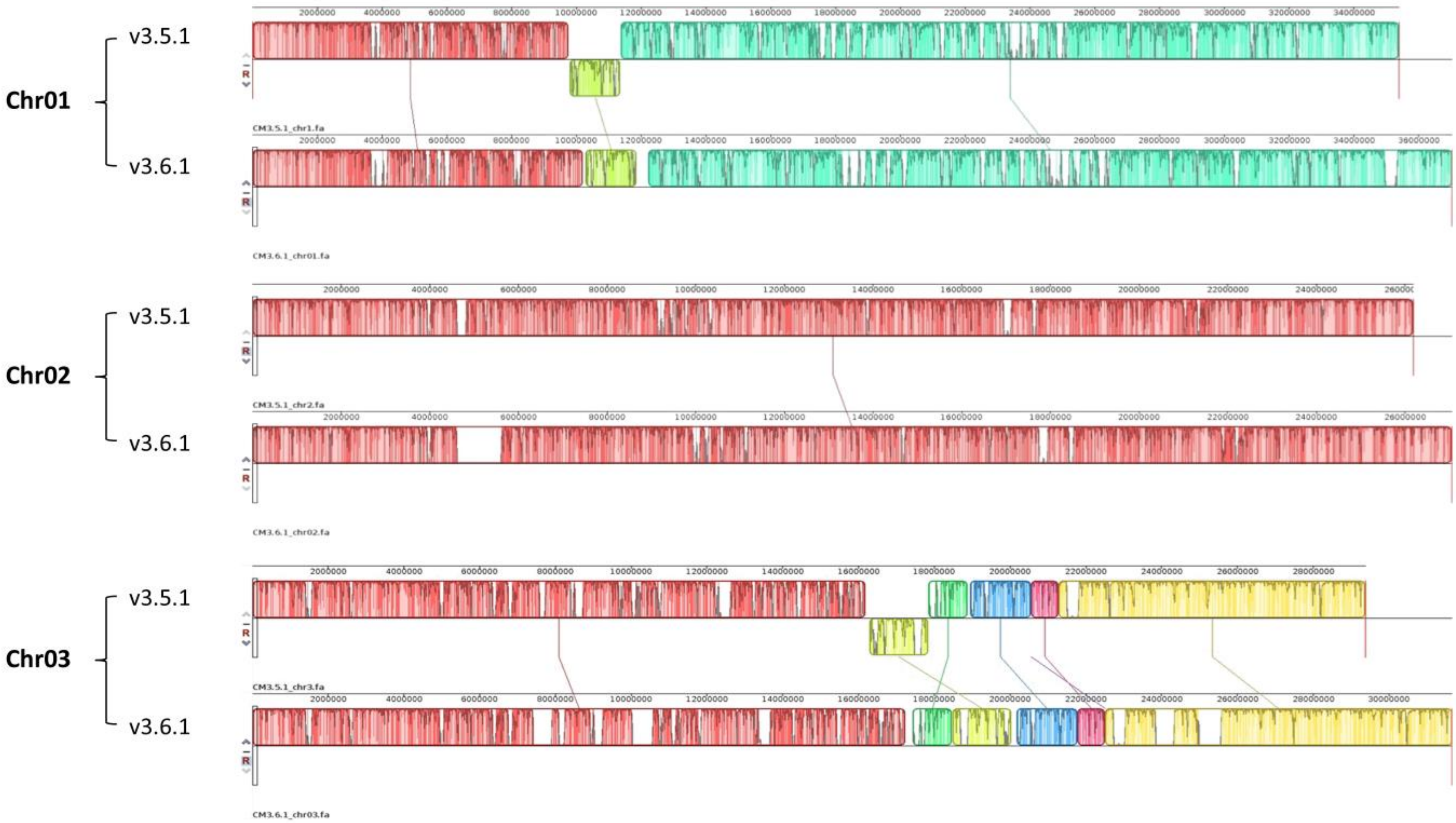
Chr01
v3.5.1
v3.6.1
CM3.5.1_chr1.fa
CM3.6.1_chr01.fa
Chr02
v3.5.1
v3.6.1
CM3.5.1_chr2.fa
CM3.6.1_chr02.fa
Chr03
v3.5.1
v3.6.1
CM3.5.1_chr3.fa
CM3.6.1_chr03.fa

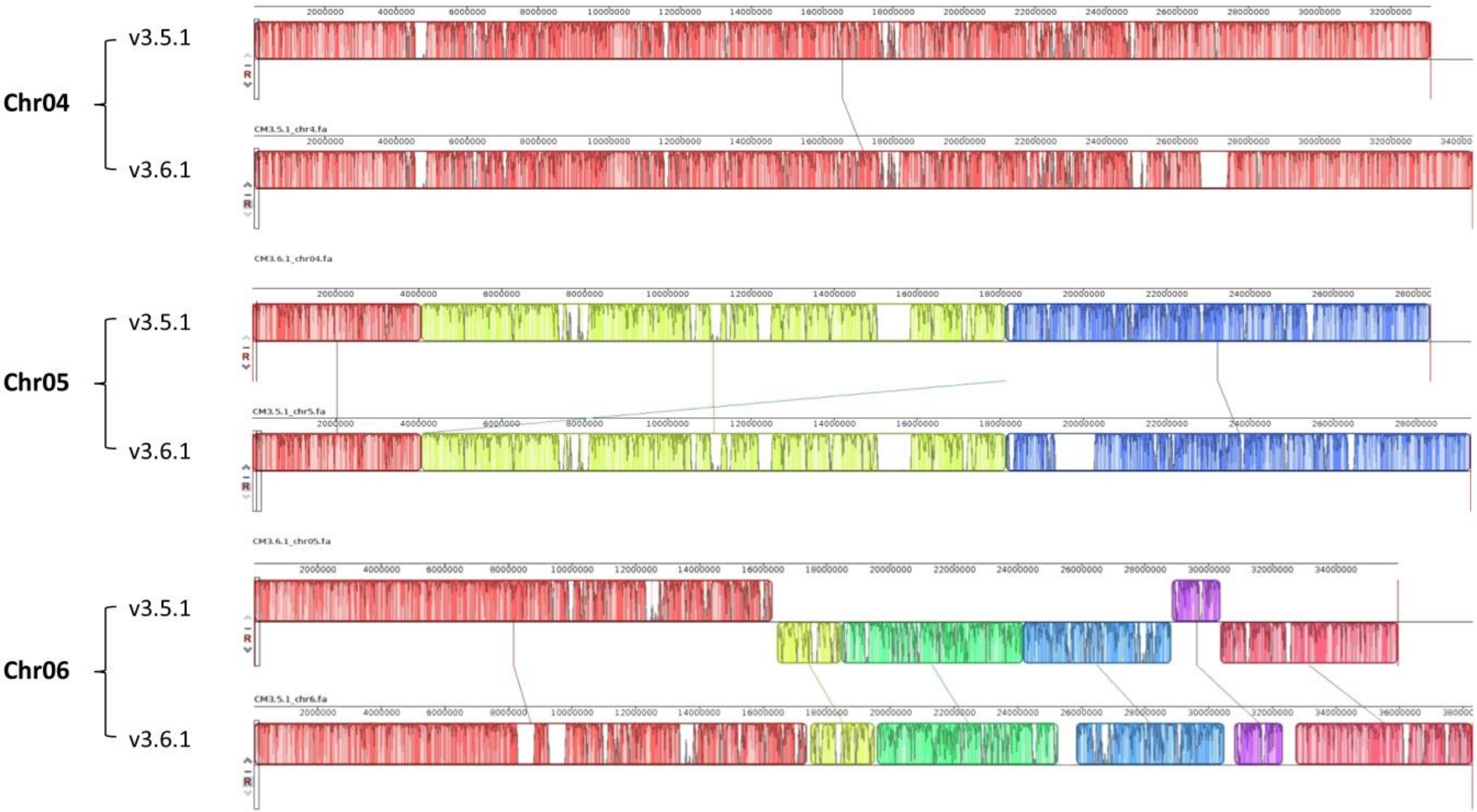
Chr04
v3.5.1
v3.6.1
CM3.5.1_chr4.fa
CM3.6.1_chr04.fa
Chr05
v3.5.1
v3.6.1
CM3.5.1_chr5.fa
CM3.6.1_chr05.fa
Chr06
v3.5.1
v3.6.1
CM3.5.1_chr6.fa

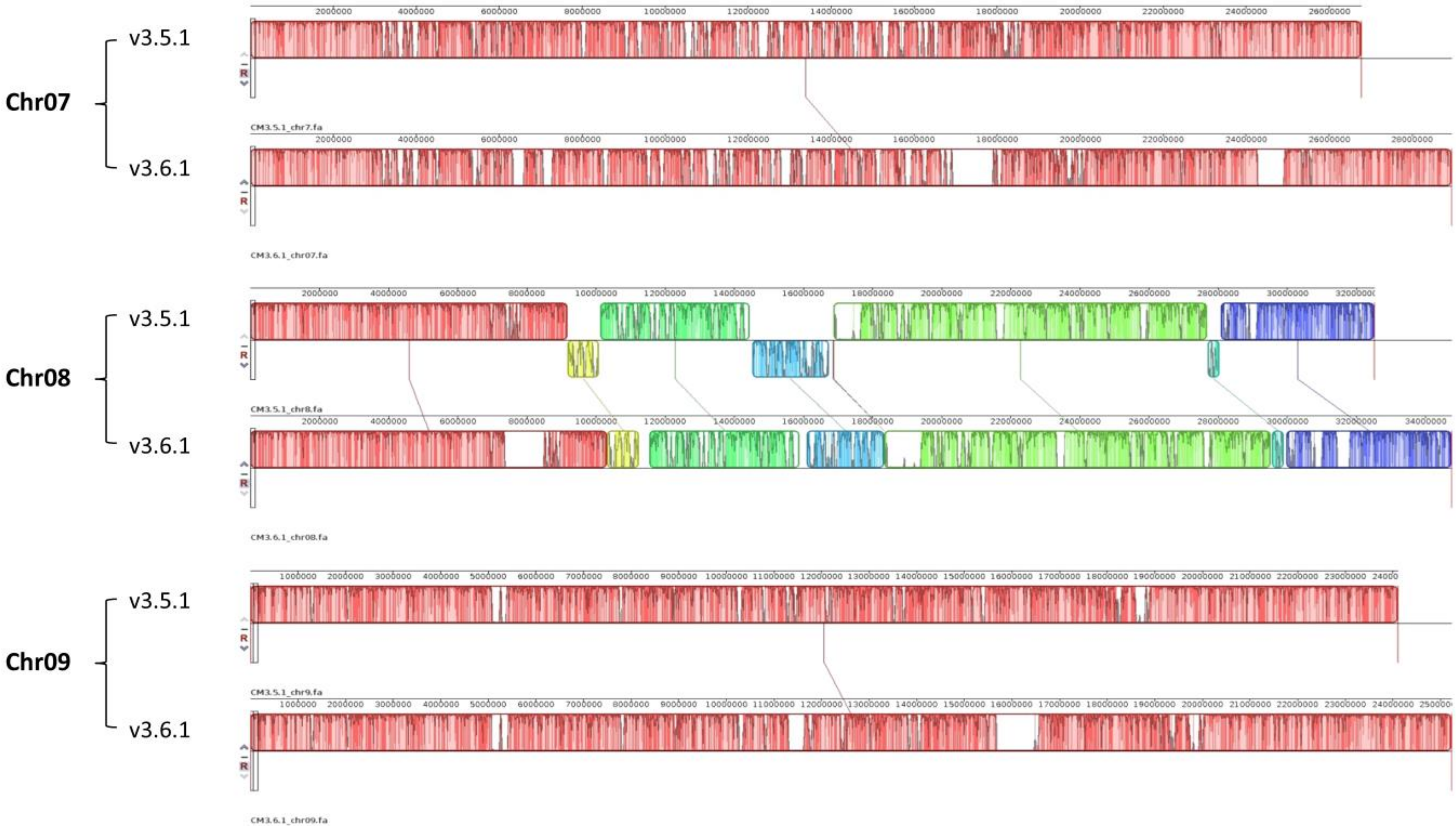
Chr07
v3.5.1
v3.6.1
CM3.5.1_chr7.fa
CM3.6.1_chr07.fa
Chr08
v3.5.1
v3.6.1
CM3.5.1_chr8.fa
CM3.6.1_chr08.fa
Chr09
v3.5.1
v3.6.1
CM3.5.1_chr9.fa
CM3.6.1_chr09.fa

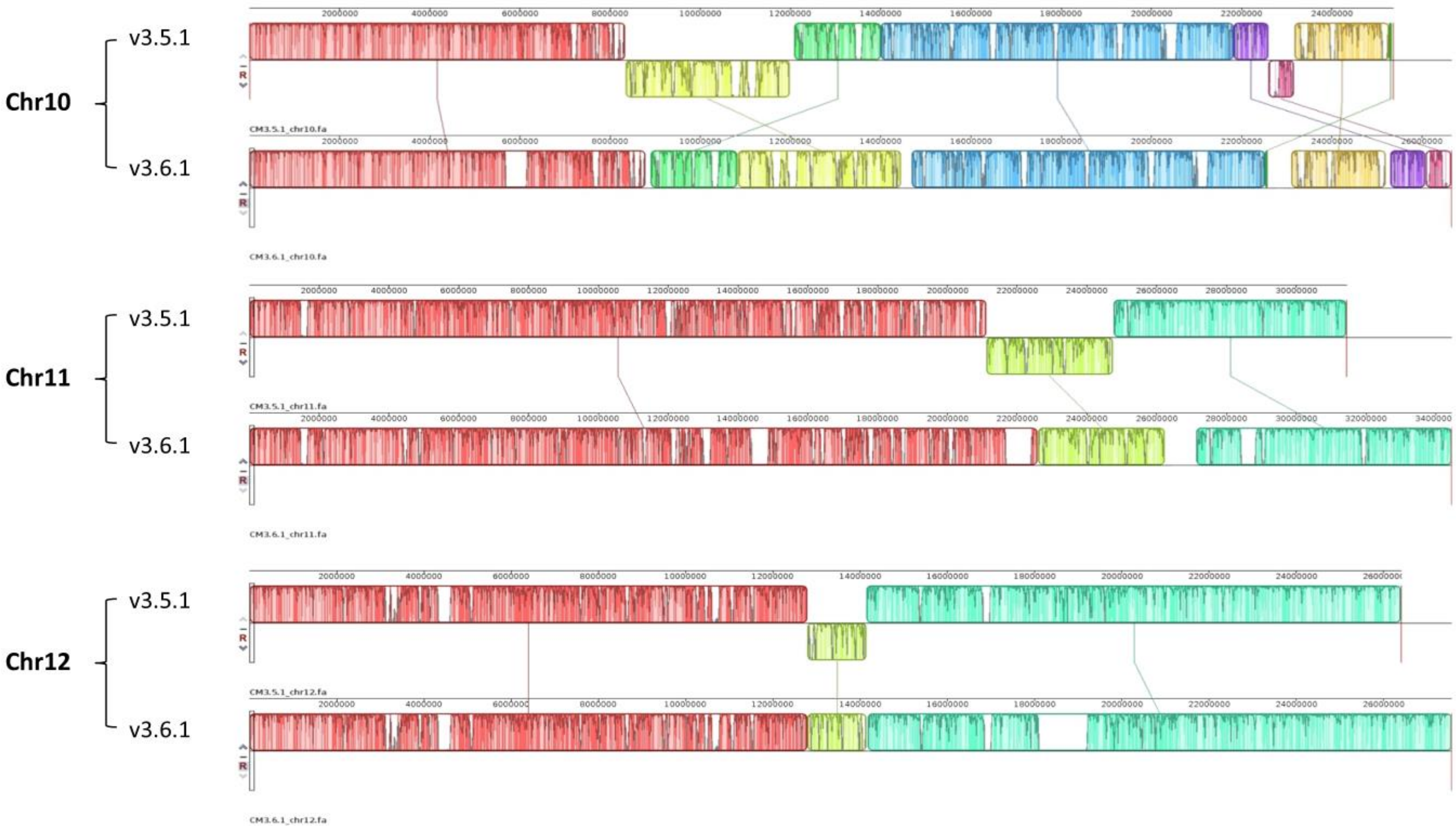
Chr10
v3.5.1
v3.6.1
CM3.5.1_chr10.fa
CM3.6.1_chr10.fa
Chr11
v3.5.1
v3.6.1
CM3.5.1_chr11.fa
CM3.6.1_chr11.fa
Chr12
v3.5.1
v3.6.1
CM3.5.1_chr12.fa
CM3.6.1_chr12.fa

Supplementary Figure S2. The workflow used for the melon v4.0 genome annotation.

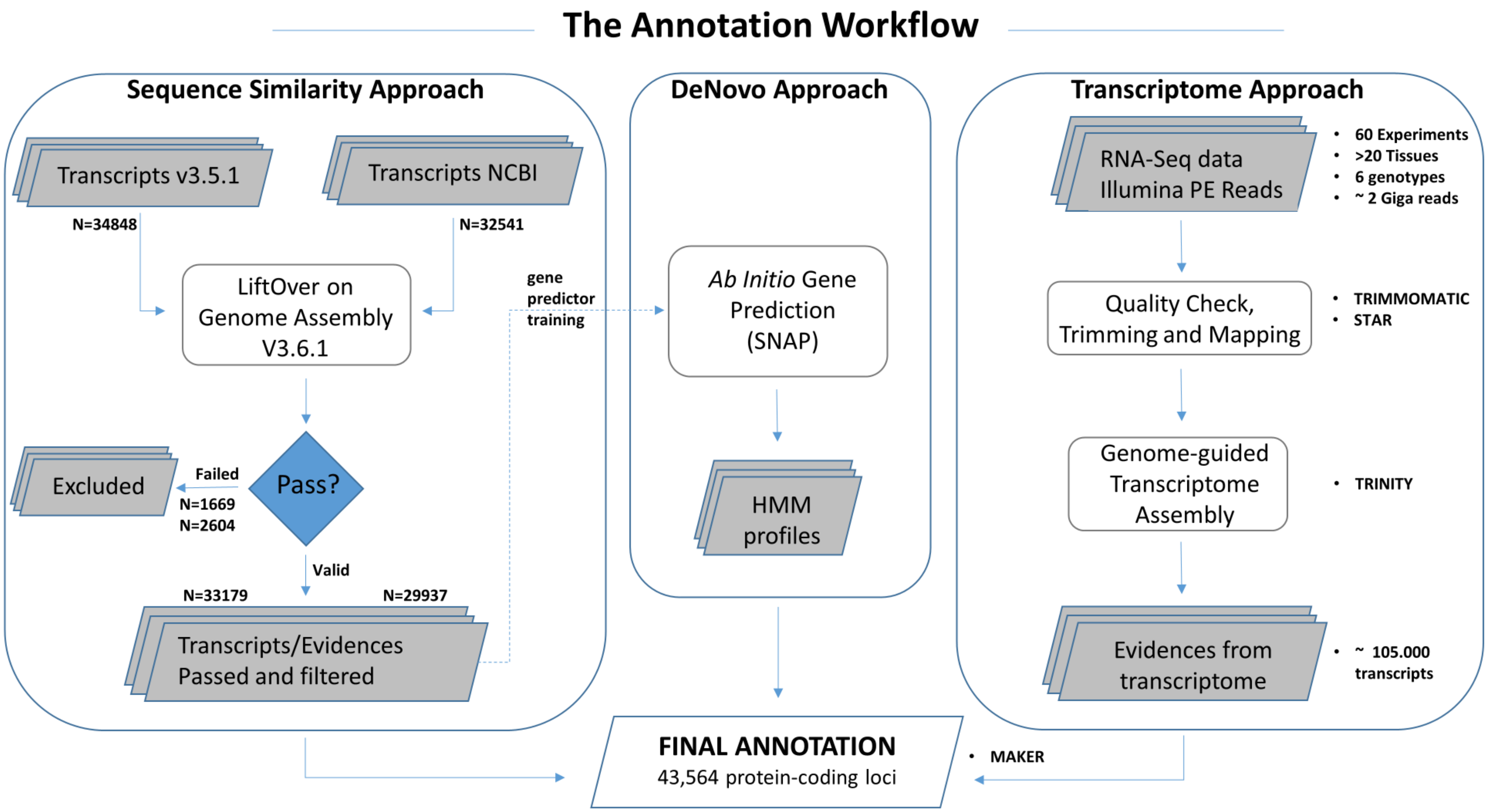

Supplementary Figure S3. Examples of improved gene annotation in melon v4.0 in comparison with the previous annotation (v3.5.1). In both the annotation tracks (v4.0 and v3.5.1) yellow boxes represent CDS, white boxes UTR and black lines introns. In the RNA-Seq track, the coloured lines (light and dark red and light and dark blue) represent mapped reads and grey lines link the split part of the reads (denoting correctness of intron/exon definition).

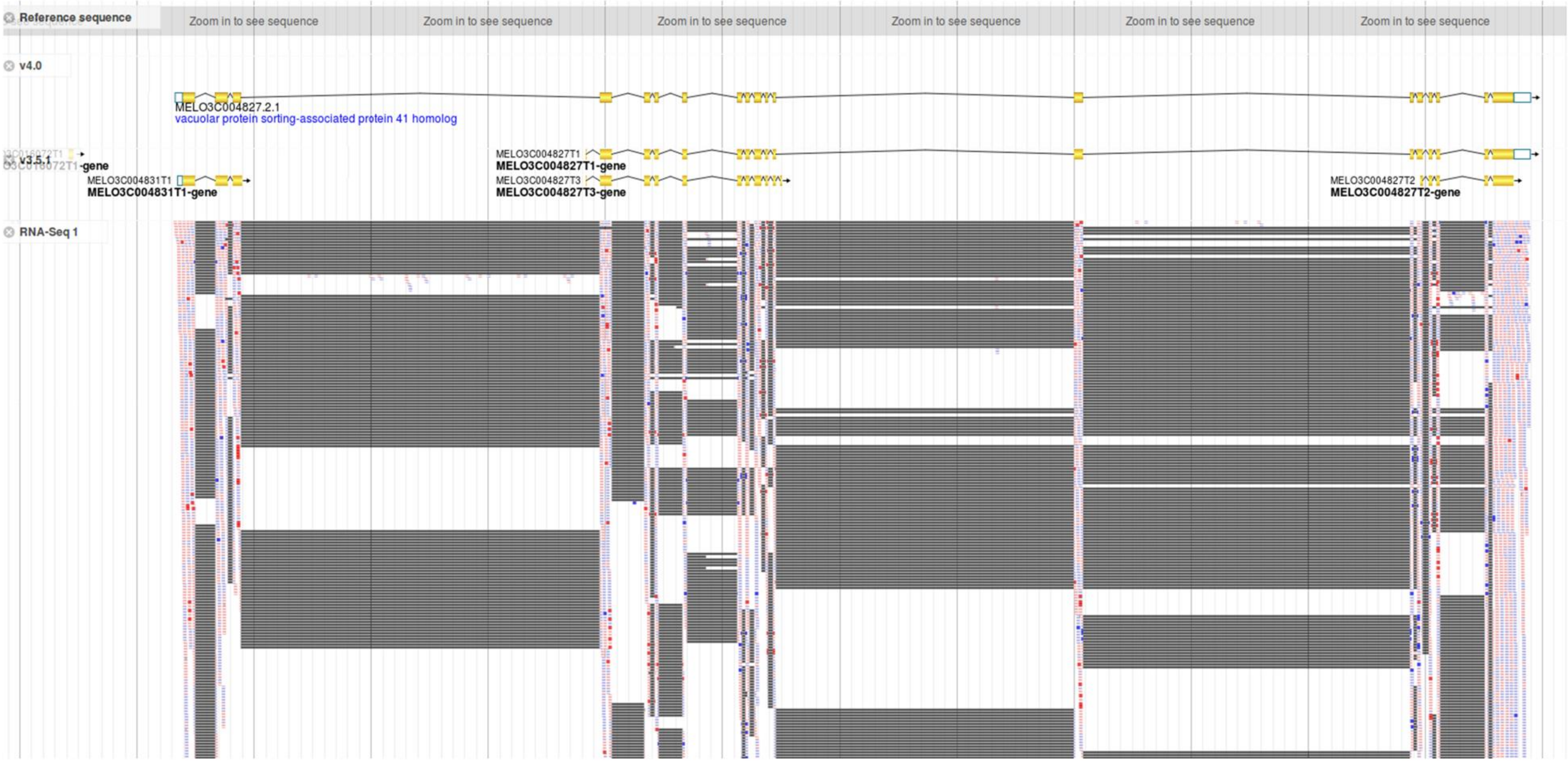
Reference sequence
Zoom in to see sequence
Zoom in to see sequence
Zoom in to see sequence
Zoom in to see sequence
Zoom in to see sequence
Zoom in to see sequence
v4.0
MELO3C004827.2.1
vacuolar protein sorting-associated protein 41 homolog
v3.5.1
MELO3C004831T1
MELO3C004831T1-gene
MELO3C004827T1
MELO3C004827T1-gene
MELO3C004827T3
MELO3C004827T3-gene
MELO3C004827T2
MELO3C004827T2-gene
RNA-Seq 1

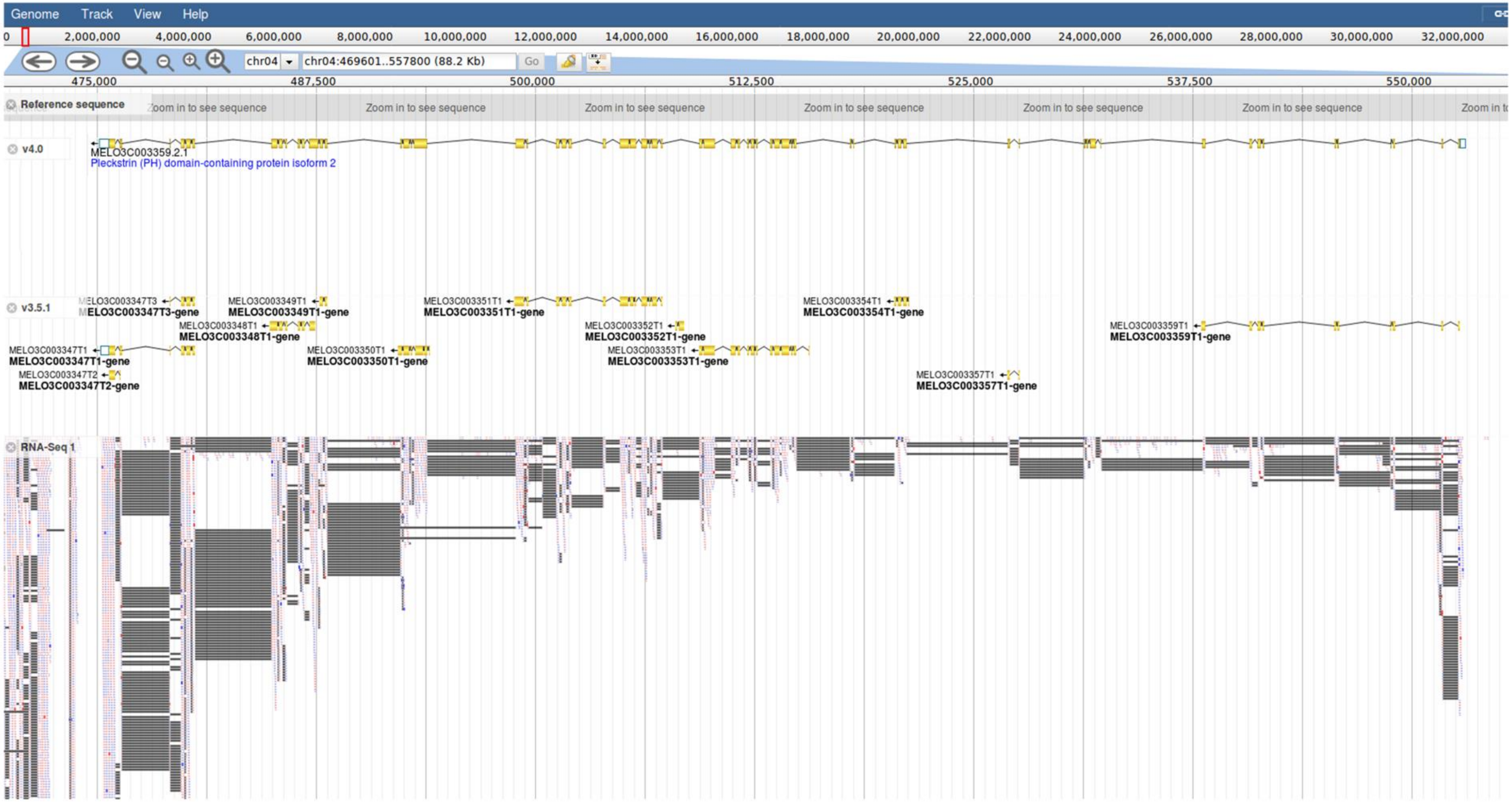
Genome
Track
View
Help
chr04
chr04:469601..557800 (88.2 Kb)
Go
Reference sequence
Zoom in to see sequence
v4.0
MELO3C003359.2.1
Pleckstrin (PH) domain-containing protein isoform 2
v3.5.1
MELO3C003347T3-gene
MELO3C003349T1-gene
MELO3C003351T1-gene
MELO3C003354T1-gene
MELO3C003348T1-gene
MELO3C003352T1-gene
MELO3C003359T1-gene
MELO3C003347T1-gene
MELO3C003350T1-gene
MELO3C003353T1-gene
MELO3C003347T2-gene
MELO3C003357T1-gene
RNA-Seq 1

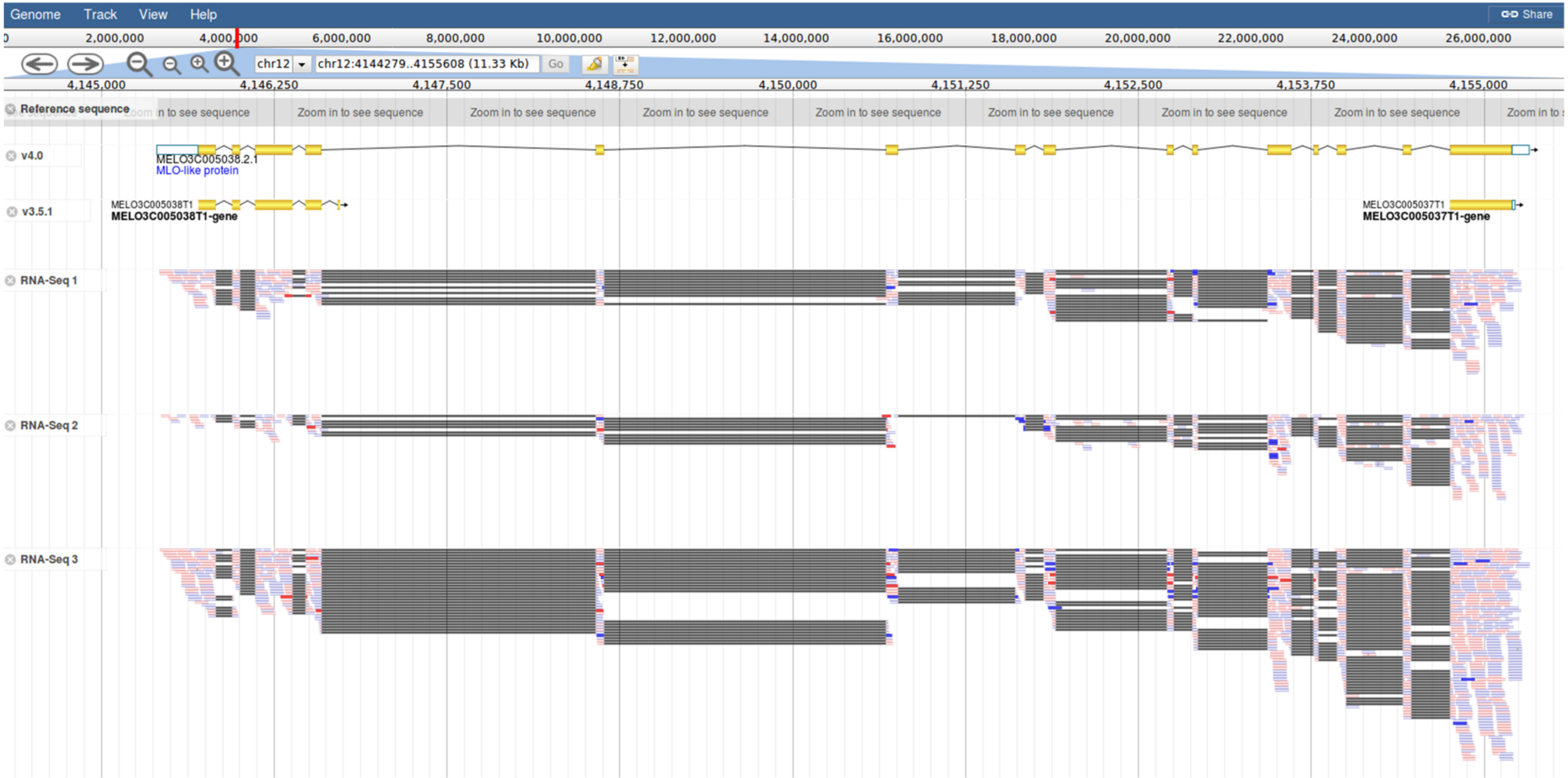
Genome
Track
View
Help
Share
2,000,000
4,000,000
6,000,000
8,000,000
10,000,000
12,000,000
14,000,000
16,000,000
18,000,000
20,000,000
22,000,000
24,000,000
26,000,000
chr12
chr12:4144279..4155608 (11.33 Kb)
Go
4,145,000
4,146,250
4,147,500
4,148,750
4,150,000
4,151,250
4,152,500
4,153,750
4,155,000
Reference sequence
Zoom in to see sequence
v4.0
MELO3C005038.2.1
MLO-like protein
v3.5.1
MELO3C005038T1
MELO3C005038T1-gene
MELO3C005037T1
MELO3C005037T1-gene
RNA-Seq 1
RNA-Seq 2
RNA-Seq 3

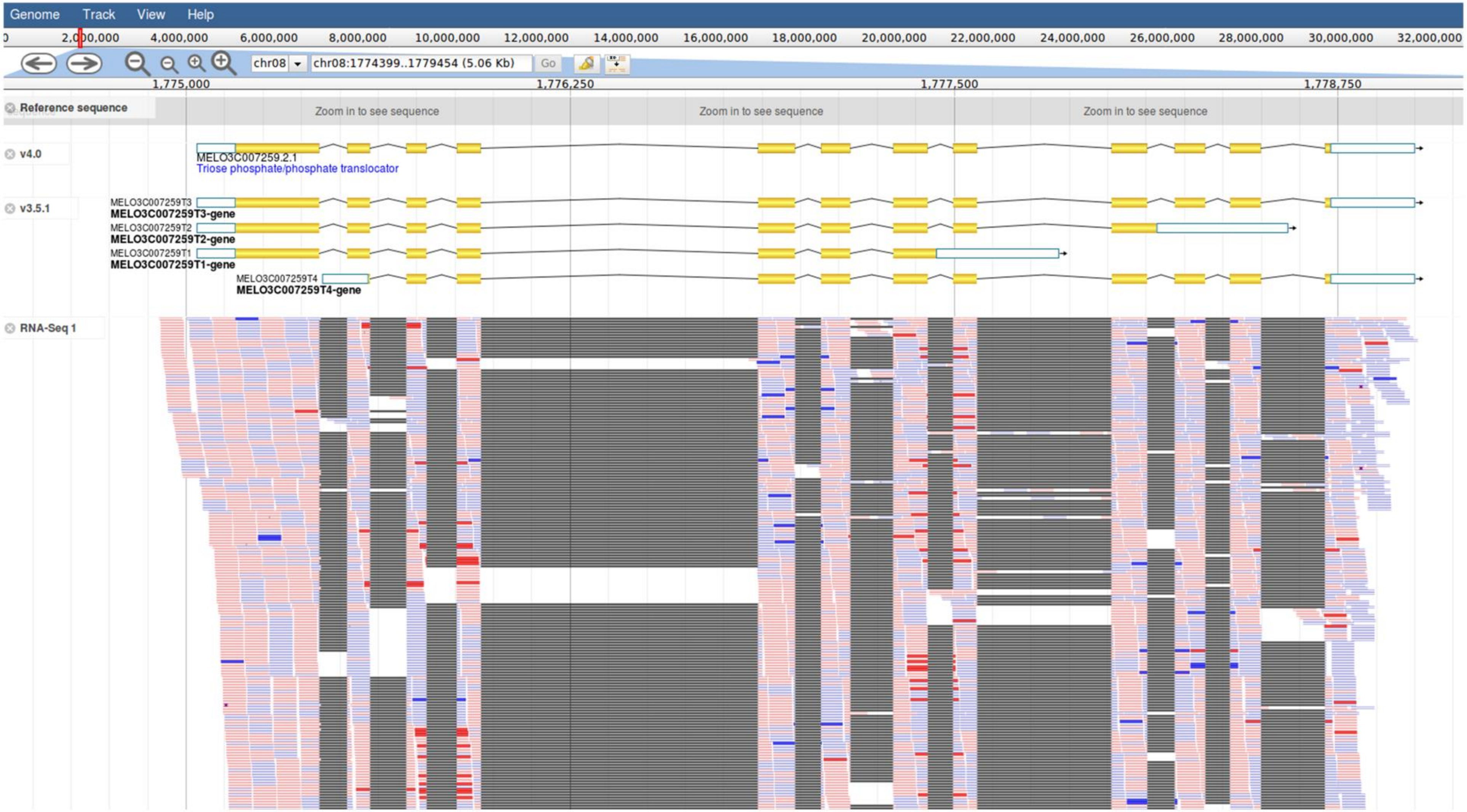
Genome Track View Help
chr08
chr08:1774399..1779454 (5.06 Kb)
Go
1,775,000
1,776,250
1,777,500
1,778,750
Reference sequence
Zoom in to see sequence
v4.0
MELO3C007259.2.1
Triose phosphate/phosphate translocator
v3.5.1
MELO3C007259T3
MELO3C007259T3-gene
MELO3C007259T2
MELO3C007259T2-gene
MELO3C007259T1
MELO3C007259T1-gene
MELO3C007259T4
MELO3C007259T4-gene
RNA-Seq 1

Supplementary Tables

Supplementary Table S1. The optical assembly. For each of the 27 superscaffolds ("optical assembly"), the ID, the start and the end position as well as the orientation of each of the scaffolds used to build it are shown.

Superscaffold_ID	Source	Type	Scaffold_ID	scaffold_start	scaffold_end	Orientation
superscaffold1	OpGen_GenomeBuilder	supercontig	CM3.6.1_SCAFFOLD00046	1	3263061	-
superscaffold1	OpGen_GenomeBuilder	supercontig	CM3.6.1_SCAFFOLD00017	3375831	9705174	-
superscaffold1	OpGen_GenomeBuilder	supercontig	CM3.6.1_SCAFFOLD00019	10188391	16144268	-
superscaffold1	OpGen_GenomeBuilder	supercontig	CM3.6.1_SCAFFOLD00072	16515452	18099981	+
superscaffold1	OpGen_GenomeBuilder	supercontig	CM3.6.1_SCAFFOLD00065	18116864	20057933	-
superscaffold1	OpGen_GenomeBuilder	supercontig	CM3.6.1_SCAFFOLD00061	20169503	22295222	+
superscaffold1	OpGen_GenomeBuilder	supercontig	CM3.6.1_SCAFFOLD00066	22460056	24356095	+
superscaffold1	OpGen_GenomeBuilder	supercontig	CM3.6.1_SCAFFOLD00034	24578339	28371817	-
superscaffold2	OpGen_GenomeBuilder	supercontig	CM3.6.1_SCAFFOLD00052	1	2473091	-
superscaffold2	OpGen_GenomeBuilder	supercontig	CM3.6.1_SCAFFOLD00047	2606643	5375121	+
superscaffold2	OpGen_GenomeBuilder	supercontig	CM3.6.1_SCAFFOLD00115	5719146	5924875	+
superscaffold2	OpGen_GenomeBuilder	supercontig	CM3.6.1_SCAFFOLD00082	6062381	7319030	-
superscaffold2	OpGen_GenomeBuilder	supercontig	CM3.6.1_SCAFFOLD00039	8211214	11866580	+
superscaffold3	OpGen_GenomeBuilder	supercontig	CM3.6.1_SCAFFOLD00053	1	2397303	-
superscaffold3	OpGen_GenomeBuilder	supercontig	CM3.6.1_SCAFFOLD00108	2397304	2757177	+
superscaffold3	OpGen_GenomeBuilder	supercontig	CM3.6.1_SCAFFOLD00041	2966745	6623346	-
superscaffold3	OpGen_GenomeBuilder	supercontig	CM3.6.1_SCAFFOLD00075	7026234	8649655	-
superscaffold3	OpGen_GenomeBuilder	supercontig	CM3.6.1_SCAFFOLD00097	8668478	9257469	-
superscaffold3	OpGen_GenomeBuilder	supercontig	CM3.6.1_SCAFFOLD00063	9277726	11307007	-

superscaffold3	OpGen_GenomeBuilder	supercontig	CM3.6.1_SCAFFOLD00016	11789426	17455488	+
superscaffold4	OpGen_GenomeBuilder	supercontig	CM3.6.1_SCAFFOLD00040	1	3616973	-
superscaffold4	OpGen_GenomeBuilder	supercontig	CM3.6.1_SCAFFOLD00096	4077861	4729230	+
superscaffold4	OpGen_GenomeBuilder	supercontig	CM3.6.1_SCAFFOLD00014	5322457	11428856	-
superscaffold5	OpGen_GenomeBuilder	supercontig	CM3.6.1_SCAFFOLD00079	1	1368188	-
superscaffold5	OpGen_GenomeBuilder	supercontig	CM3.6.1_SCAFFOLD00048	1401078	4187334	-
superscaffold5	OpGen_GenomeBuilder	supercontig	CM3.6.1_SCAFFOLD00051	4191110	6704145	+
superscaffold6	OpGen_GenomeBuilder	supercontig	CM3.6.1_SCAFFOLD00107	1	368707	+
superscaffold6	OpGen_GenomeBuilder	supercontig	CM3.6.1_SCAFFOLD00086	419718	1574368	+
superscaffold6	OpGen_GenomeBuilder	supercontig	CM3.6.1_SCAFFOLD00076	1578484	3132331	+
superscaffold6	OpGen_GenomeBuilder	supercontig	CM3.6.1_SCAFFOLD00010	3219465	10750746	+
superscaffold6	OpGen_GenomeBuilder	supercontig	CM3.6.1_SCAFFOLD00111	10760515	11020278	+
superscaffold6	OpGen_GenomeBuilder	supercontig	CM3.6.1_SCAFFOLD00058	11599372	13935368	+
superscaffold6	OpGen_GenomeBuilder	supercontig	CM3.6.1_SCAFFOLD00036	14234008	18409549	-
superscaffold6	OpGen_GenomeBuilder	supercontig	CM3.6.1_SCAFFOLD01602	18685769	19605311	+
superscaffold7	OpGen_GenomeBuilder	supercontig	CM3.6.1_SCAFFOLD00109	1	410055	-
superscaffold7	OpGen_GenomeBuilder	supercontig	CM3.6.1_SCAFFOLD00084	622147	1762413	+
superscaffold7	OpGen_GenomeBuilder	supercontig	CM3.6.1_SCAFFOLD00020	1775120	7504981	-
superscaffold7	OpGen_GenomeBuilder	supercontig	CM3.6.1_SCAFFOLD00085	7980006	9143592	+
superscaffold7	OpGen_GenomeBuilder	supercontig	CM3.6.1_SCAFFOLD00037	9361512	13477577	-
superscaffold7	OpGen_GenomeBuilder	supercontig	CM3.6.1_SCAFFOLD00070	13477578	15204175	-
superscaffold7	OpGen_GenomeBuilder	supercontig	CM3.6.1_SCAFFOLD00050	15204176	17846088	+

superscaffold7	OpGen_GenomeBuilder	supercontig	CM3.6.1_SCAFFOLD00045	17942791	20210606	-
superscaffold7	OpGen_GenomeBuilder	supercontig	CM3.6.1_SCAFFOLD00059	20258565	22350097	-
superscaffold8	OpGen_GenomeBuilder	supercontig	CM3.6.1_SCAFFOLD00087	1	1036637	+
superscaffold8	OpGen_GenomeBuilder	supercontig	CM3.6.1_SCAFFOLD00080	1065740	2425425	+
superscaffold8	OpGen_GenomeBuilder	supercontig	CM3.6.1_SCAFFOLD00004	2425426	11594477	+
superscaffold9	OpGen_GenomeBuilder	supercontig	CM3.6.1_SCAFFOLD00021	1	5562797	+
superscaffold9	OpGen_GenomeBuilder	supercontig	CM3.6.1_SCAFFOLD00078	5959411	7477880	+
superscaffold9	OpGen_GenomeBuilder	supercontig	CM3.6.1_SCAFFOLD00028	7806354	12501152	-
superscaffold9	OpGen_GenomeBuilder	supercontig	CM3.6.1_SCAFFOLD00023	13016850	18713093	-
superscaffold9	OpGen_GenomeBuilder	supercontig	CM3.6.1_SCAFFOLD00062	18754328	20813568	+
superscaffold9	OpGen_GenomeBuilder	supercontig	CM3.6.1_SCAFFOLD00056	21805886	23893126	+
superscaffold9	OpGen_GenomeBuilder	supercontig	CM3.6.1_SCAFFOLD00094	23999181	24767125	+
superscaffold9	OpGen_GenomeBuilder	supercontig	CM3.6.1_SCAFFOLD00101	24767939	25356353	-
superscaffold10	OpGen_GenomeBuilder	supercontig	CM3.6.1_SCAFFOLD00011	1	6910996	+
superscaffold10	OpGen_GenomeBuilder	supercontig	CM3.6.1_SCAFFOLD00098	7684809	8260478	+
superscaffold10	OpGen_GenomeBuilder	supercontig	CM3.6.1_SCAFFOLD00104	8698670	9175806	-
superscaffold10	OpGen_GenomeBuilder	supercontig	CM3.6.1_SCAFFOLD00064	9504379	11516288	+
superscaffold11	OpGen_GenomeBuilder	supercontig	CM3.6.1_SCAFFOLD00008	1	7389538	+
superscaffold11	OpGen_GenomeBuilder	supercontig	CM3.6.1_SCAFFOLD01598	7897253	10021805	-
superscaffold11	OpGen_GenomeBuilder	supercontig	CM3.6.1_SCAFFOLD00015	10563123	17253475	-
superscaffold11	OpGen_GenomeBuilder	supercontig	CM3.6.1_SCAFFOLD00088	17432759	18476739	+
superscaffold11	OpGen_GenomeBuilder	supercontig	CM3.6.1_SCAFFOLD00073	18476740	20125217	+

superscaffold12	OpGen_GenomeBuilder	supercontig	CM3.6.1_SCAFFOLD00091	1	957300	+
superscaffold12	OpGen_GenomeBuilder	supercontig	CM3.6.1_SCAFFOLD00022	2011753	6904431	+
superscaffold13	OpGen_GenomeBuilder	supercontig	CM3.6.1_SCAFFOLD00018	1	6010539	+
superscaffold13	OpGen_GenomeBuilder	supercontig	CM3.6.1_SCAFFOLD00083	6010540	7305435	-
superscaffold13	OpGen_GenomeBuilder	supercontig	CM3.6.1_SCAFFOLD00054	7310535	9781400	+
superscaffold13	OpGen_GenomeBuilder	supercontig	CM3.6.1_SCAFFOLD00102	9824497	10364267	-
superscaffold13	OpGen_GenomeBuilder	supercontig	CM3.6.1_SCAFFOLD00100	10367202	11006610	+
superscaffold14	OpGen_GenomeBuilder	supercontig	CM3.6.1_SCAFFOLD00071	1	1861257	+
superscaffold14	OpGen_GenomeBuilder	supercontig	CM3.6.1_SCAFFOLD00035	2849157	6915873	-
superscaffold14	OpGen_GenomeBuilder	supercontig	CM3.6.1_SCAFFOLD00090	6946630	7895742	+
superscaffold14	OpGen_GenomeBuilder	supercontig	CM3.6.1_SCAFFOLD00013	7909968	12373395	+
superscaffold14	OpGen_GenomeBuilder	supercontig	CM3.6.1_SCAFFOLD00092	13281809	14113970	-
superscaffold14	OpGen_GenomeBuilder	supercontig	CM3.6.1_SCAFFOLD00081	14229850	15517971	+
superscaffold15	OpGen_GenomeBuilder	supercontig	CM3.6.1_SCAFFOLD00009	1	8131835	+
superscaffold15	OpGen_GenomeBuilder	supercontig	CM3.6.1_SCAFFOLD00003	9100269	18161269	+
superscaffold16	OpGen_GenomeBuilder	supercontig	CM3.6.1_SCAFFOLD01595	1	4632491	+
superscaffold16	OpGen_GenomeBuilder	supercontig	CM3.6.1_SCAFFOLD00055	4918159	7350401	+
superscaffold17	OpGen_GenomeBuilder	supercontig	CM3.6.1_SCAFFOLD00074	1	1606365	-
superscaffold17	OpGen_GenomeBuilder	supercontig	CM3.6.1_SCAFFOLD00005	2415170	11186730	+

superscaffold18	OpGen_GenomeBuilder	supercontig	CM3.6.1_SCAFFOLD00069	1	1728327	-
superscaffold18	OpGen_GenomeBuilder	supercontig	CM3.6.1_SCAFFOLD00077	1745930	3233084	-
superscaffold18	OpGen_GenomeBuilder	supercontig	CM3.6.1_SCAFFOLD00099	3255288	3847206	+
superscaffold18	OpGen_GenomeBuilder	supercontig	CM3.6.1_SCAFFOLD00012	3868586	10570176	-
superscaffold19	OpGen_GenomeBuilder	supercontig	CM3.6.1_SCAFFOLD00060	1	1994139	-
superscaffold19	OpGen_GenomeBuilder	supercontig	CM3.6.1_SCAFFOLD00067	2347080	4090582	+
superscaffold20	OpGen_GenomeBuilder	supercontig	CM3.6.1_SCAFFOLD01600	1	532338	+
superscaffold20	OpGen_GenomeBuilder	supercontig	CM3.6.1_SCAFFOLD00002	899571	3869460	+
superscaffold21	OpGen_GenomeBuilder	supercontig	CM3.6.1_SCAFFOLD00007	1	7340689	+
superscaffold21	OpGen_GenomeBuilder	supercontig	CM3.6.1_SCAFFOLD00068	8492010	10329996	-
superscaffold22	OpGen_GenomeBuilder	supercontig	CM3.6.1_SCAFFOLD00027	1	4438709	+
superscaffold22	OpGen_GenomeBuilder	supercontig	CM3.6.1_SCAFFOLD00031	5090048	9142449	+
superscaffold23	OpGen_GenomeBuilder	supercontig	CM3.6.1_SCAFFOLD00001	1	8359259	+
superscaffold23	OpGen_GenomeBuilder	supercontig	CM3.6.1_SCAFFOLD00049	9490028	12342704	-
superscaffold24	OpGen_GenomeBuilder	supercontig	CM3.6.1_SCAFFOLD00006	1	8259204	+
superscaffold24	OpGen_GenomeBuilder	supercontig	CM3.6.1_SCAFFOLD00038	9784023	13532388	+
superscaffold25	OpGen_GenomeBuilder	supercontig	CM3.6.1_SCAFFOLD00033	1	4162015	-
superscaffold25	OpGen_GenomeBuilder	supercontig	CM3.6.1_SCAFFOLD00024	4409441	10078632	+
superscaffold25	OpGen_GenomeBuilder	supercontig	CM3.6.1_SCAFFOLD00025	10880865	15567957	-

superscaffold26	OpGen_GenomeBuilder	supercontig	CM3.6.1_SCAFFOLD00089	1	921585	+
superscaffold26	OpGen_GenomeBuilder	supercontig	CM3.6.1_SCAFFOLD00030	929822	5217847	+
superscaffold27	OpGen_GenomeBuilder	supercontig	CM3.6.1_SCAFFOLD00105	1	461745	+
superscaffold27	OpGen_GenomeBuilder	supercontig	CM3.6.1_SCAFFOLD00057	476999	2731682	+

Supplementary Table S2. Detailed comparison between v3.5.1 and v3.6.1 assemblies (including chromosome 0). For each chromosome the length, the number of Ns as well as the ratio between the two releases and the details of the improvement achieved are reported.

chromosome	V3.5.1		V3.6.1		Ratio (3.6.1 vs 3.5.1)		Improvements / Comments
	length (bp)	number of Ns (bp)	length (bp)	number of Ns (bp)	length	number of Ns	
Chr00	51.963.947	*32.131.059*	41.641.883	*22.464.661*	-19,86%	-30,08%	-
Chr01	35.383.099	*4.224.098*	37.037.532	*5.746.973*	4,68%	36,05%	orientation established for 1 scaffold
Chr02	26.193.771	*2.467.907*	27.064.691	*3.272.777*	3,32%	32,61%	Complete agreement of OpGen assembly with 3.5.1 anchoring, in terms of scaffold ordering and orientation, except from CM3.5.1_SCAFFOLD00033, anchored by OpGen, which was maintained in chr10.
Chr03	29.387.469	*3.090.555*	31.666.927	*5.286.237*	7,76%	71,04%	orientation established for 1 scaffold; correction of orientation for 1 scaffold
Chr04	33.123.230	*3.484.828*	34.318.044	*4.557.078*	3,61%	30,77%	orientation established for 2 scaffolds
Chr05	28.337.775	*3.556.980*	29.324.171	*4.428.209*	3,48%	24,49%	orientation established for 2 scaffolds; pairing of scaffolds CM3.5.1_SCAFFOLD00022 and CM3.5.1_SCAFFOLD00091 found by OpGen is erroneous. Scaffold CM3.5.1_SCAFFOLD00022 should be cut at the beginning and the excised sequence to be added to CM3.5.1_SCAFFOLD01600, which previously formed part of CM3.5.1_SCAFFOLD00022.
Chr06	35.939.859	*4.056.829*	38.297.372	*6.313.150*	6,56%	55,62%	orientation established for 1 scaffold; correction of orientation for 4 scaffolds

Chr07	26.773.857	*2.703.377*	28.958.359	*4.821.595*	8,16%	78,35%	Complete agreement of OpGen assembly with 3.5.1 anchoring, in terms of scaffold ordering and orientation, expect from the maintenance of 6 scaffolds that are present in the anchoring of the 3.5.1 assembly.
Chr08	32.513.408	*3.874.538*	34.765.488	*5.785.898*	6,93%	49,33%	orientation established for 4 scaffolds; CM3.5.1_SCAFFOLD00111 wad added by OpGen; Gap size between CM3.5.1_SCAFFOLD00058 and CM3.5.1_SCAFFOLD00111, as established by OpGen, equals the size of CM3.5.1_SCAFFOLD00103 (579,514bp), which was not added by OpGen (**gap closure**)
Chr09	24.107.567	*2.533.680*	25.243.276	*3.572.934*	4,71%	41,02%	Complete agreement of OpGen assembly with 3.5.1 anchoring, in terms of scaffold ordering and orientation.
Chr10	25.362.315	*2.705.038*	26.663.822	*3.957.926*	5,13%	46,32%	CM3.5.1_SCAFFOLD00133 was removed from the pseudomolecule; orientation established for 4 scaffolds; scaffold reordering was applied, based on OpGen anchoring; The last 3 scaffolds of Superscaffold 9 from OpGen [CM3.5.1_SCAFFOLD00(056,094,101)] are joined to scaffolds that are anchored onto chr06, based on the anchoring of the 3.5.1 assembly. To break this superscaffold, CM3.5.1_SCAFFOLD00056 should have its size adjusted.
Chr11	31.442.130	*3.210.864*	34.457.057	*5.604.798*	9,59%	74,56%	Two scaffolds are added by OpGen: CM3.5.1_SCAFFOLD00109 (410,055 bp), CM3.5.1_SCAFFOLD00115 (205,730 bp)
Chr12	26.400.393	*2.792.021*	27.563.660	*3.864.732*	4,41%	38,42%	orientation established for 1 scaffold
**Total**	**406.928.820**	***70.831.774***	**417.002.282**	***79.676.968***	**2,48%**	**12,49%**	

chromosome	Non-anchored scaffolds by OpGen		Total size (bp)
	small (<=200Kb)	Large (>200Kb)	
chr01	3	1	4746010
chr02	1	-	115151
chr03	1	-	110854
chr04	5	4	12185193
chr05	-	1	3535541
chr06	3	3	4390725
chr07	4	2	4814306
chr08	2	-	823240
chr09	-	-	-
chr10	2	2	1382066
chr11	1	1	404713
chr12	-	2	3623479

Supplementary Table S3. RNA-Seq dataset used for the melon Genome-guided Transcriptome Assembly. For each experiment the sample_ID, the species, the cultivar, the tissue, the instrument used, the library layout as well as the number of spots, the average length (sum of both reads) of the reads and the reference source are reported.

Sequential _ ID	Sample_ID	Species	Cultivar	Tissue	Instrument	Library Layout	Spots No	Avg. lenght	Note
1	DRR106503	Cucumis melo	Harukei-3	callus	Illumina-HISeq 2500	Paired-end	12066215	250	Yano et al., 2017
2	DRR106504	Cucumis melo	Harukei-3	DAF15d_epicarp	Illumina-HISeq 2500	Paired-end	6025652	250	Yano et al., 2017
3	DRR106505	Cucumis melo	Harukei-3	DAF15d_flesh	Illumina-HISeq 2500	Paired-end	6192483	250	Yano et al., 2017
4	DRR106506	Cucumis melo	Harukei-3	DAF22d_epicarp	Illumina-HISeq 2500	Paired-end	5704958	250	Yano et al., 2017
5	DRR106507	Cucumis melo	Harukei-3	DAF22d_flesh	Illumina-HISeq 2500	Paired-end	4795017	250	Yano et al., 2017
6	DRR106508	Cucumis melo	Harukei-3	DAF29d_epicarp	Illumina-HISeq 2500	Paired-end	6067040	250	Yano et al., 2017
7	DRR106509	Cucumis melo	Harukei-3	DAF29d_flesh	Illumina-HISeq 2500	Paired-end	5653802	250	Yano et al., 2017

8	DRR106510	Cucumis melo	Harukei-3	DAF2d	Illumina-HISeq 2500	Paired-end	5006766	250	Yano et al., 2017
9	DRR106511	Cucumis melo	Harukei-3	DAF36d_epicarp	Illumina-HISeq 2500	Paired-end	6838695	250	Yano et al., 2017
10	DRR106512	Cucumis melo	Harukei-3	DAF36d_flesh	Illumina-HISeq 2500	Paired-end	6515888	250	Yano et al., 2017
11	DRR106513	Cucumis melo	Harukei-3	DAF43d_epicarp	Illumina-HISeq 2500	Paired-end	6648237	250	Yano et al., 2017
12	DRR106514	Cucumis melo	Harukei-3	DAF43d_flesh	Illumina-HISeq 2500	Paired-end	6725605	250	Yano et al., 2017
13	DRR106515	Cucumis melo	Harukei-3	DAF4d	Illumina-HISeq 2500	Paired-end	4658443	250	Yano et al., 2017
14	DRR106516	Cucumis melo	Harukei-3	DAF50d_epicarp	Illumina-HISeq 2500	Paired-end	6038720	250	Yano et al., 2017
15	DRR106517	Cucumis melo	Harukei-3	DAF50d_flesh	Illumina-HISeq 2500	Paired-end	5934716	250	Yano et al., 2017
16	DRR106518	Cucumis melo	Harukei-3	DAF8d_epicarp	Illumina-HISeq 2500	Paired-end	6508237	250	Yano et al., 2017
17	DRR106519	Cucumis melo	Harukei-3	DAF8d_flesh	Illumina-HISeq 2500	Paired-end	4849932	250	Yano et al., 2017
18	DRR106520	Cucumis melo	Harukei-3	dryseed	Illumina-HISeq 2500	Paired-end	11030405	250	Yano et al., 2017

19	DRR106521	Cucumis melo	Harukei-3	female_flower_DAF0_ ovary	Illumina-HISeq 2500	Paired-end	7069462	250	Yano et al., 2017
20	DRR106522	Cucumis melo	Harukei-3	female_flower_DAF0_ petal	Illumina-HISeq 2500	Paired-end	5052377	250	Yano et al., 2017
21	DRR106523	Cucumis melo	Harukei-3	female_flower_DAF0_ stigma	Illumina-HISeq 2500	Paired-end	5571730	250	Yano et al., 2017
22	DRR106524	Cucumis melo	Harukei-3	leaves_6th	Illumina-HISeq 2500	Paired-end	4296364	250	Yano et al., 2017
23	DRR106525	Cucumis melo	Harukei-3	leaves_9th	Illumina-HISeq 2500	Paired-end	8376072	250	Yano et al., 2017
24	DRR106526	Cucumis melo	Harukei-3	leaves_12th	Illumina-HISeq 2500	Paired-end	4521114	250	Yano et al., 2017
25	DRR106527	Cucumis melo	Harukei-3	male_flower_ anther	Illumina-HISeq 2500	Paired-end	5048873	250	Yano et al., 2017
26	DRR106528	Cucumis melo	Harukei-3	root	Illumina-HISeq 2500	Paired-end	5696452	250	Yano et al., 2017
27	DRR106529	Cucumis melo	Harukei-3	stem_ middleside	Illumina-HISeq 2500	Paired-end	5402392	250	Yano et al., 2017
28	DRR106530	Cucumis melo	Harukei-3	stem_upside	Illumina-HISeq 2500	Paired-end	5823477	250	Yano et al., 2017
29	DRR106531	Cucumis melo	Harukei-3	tendril	Illumina-HISeq 2500	Paired-end	13424481	250	Yano et al., 2017

30	DRR106532	Cucumis melo	Harukei-3	young_leaves	Illumina-HISeq 2500	Paired-end	5273641	250	Yano et al., 2017
31	SRR2962681	Cucumis melo	Makuwa-KM	flower_female	Illumina-HISeq 2500	Paired-end	31237797	202	Kim et al., 2016
32	SRR2962682	Cucumis melo	Makuwa-KM	flower_male	Illumina-HISeq 2500	Paired-end	31634642	202	Kim et al., 2016
33	SRR2962683	Cucumis melo	Makuwa-KM	fruit	Illumina-HISeq 2500	Paired-end	41733461	202	Kim et al., 2016
34	SRR2962684	Cucumis melo	Makuwa-KM	leaf	Illumina-HISeq 2500	Paired-end	30296880	202	Kim et al., 2016
35	SRR2962686	Cucumis melo	Makuwa-KM	root	Illumina-HISeq 2500	Paired-end	32706926	202	Kim et al., 2016
36	SRR2962687	Cucumis melo	Makuwa-NW	flower_female	Illumina-HISeq 2500	Paired-end	24880861	202	Kim et al., 2016
37	SRR2962688	Cucumis melo	Makuwa-NW	flower_male	Illumina-HISeq 2500	Paired-end	26474098	202	Kim et al., 2016
38	SRR2962689	Cucumis melo	Makuwa-NW	fruit	Illumina-HISeq 2500	Paired-end	38991361	202	Kim et al., 2016
39	SRR2962690	Cucumis melo	Makuwa-NW	leaf	Illumina-HISeq 2500	Paired-end	48138316	202	Kim et al., 2016
40	SRR2962691	Cucumis melo	Makuwa-NW	root	Illumina-HISeq 2500	Paired-end	44123310	202	Kim et al., 2016
41	C5J7AACXX_1_14	Cucumis melo	Piel de Sapo	Flesh fruit-25 DAP	Illumina-HISeq 2500	Paired-end	39917182	150	Unpublished data. Private collection from CRAG

42	C5J7AACXX_3_15	Cucumis melo	Piel de Sapo	Flesh fruit-25 DAP	Illumina-HISeq 2500	Paired-end	29978607	150	Unpublished data. Private collection from CRAG
43	C5J82ACXX_7_13	Cucumis melo	Piel de Sapo	Flesh fruit-25 DAP	Illumina-HISeq 2500	Paired-end	28328647	150	Unpublished data. Private collection from CRAG
44	C5J64ACXX_3_19	Cucumis melo	Piel de Sapo	Flesh fruit-35 DAP	Illumina-HISeq 2500	Paired-end	28403012	150	Unpublished data. Private collection from CRAG
45	C5J82ACXX_6_18	Cucumis melo	Piel de Sapo	Flesh fruit-35 DAP	Illumina-HISeq 2500	Paired-end	22557074	150	Unpublished data. Private collection from CRAG
46	C5J64ACXX_1_20	Cucumis melo	Piel de Sapo	Flesh fruit-45 DAP	Illumina-HISeq 2500	Paired-end	29738355	150	Unpublished data. Private collection from CRAG
47	C5J64ACXX_5_22	Cucumis melo	Piel de Sapo	Flesh fruit-45 DAP	Illumina-HISeq 2500	Paired-end	32853759	150	Unpublished data. Private collection from CRAG

48	C5J82ACXX_8_21	Cucumis melo	Piel de Sapo	Flesh fruit-45 DAP	Illumina-HISeq 2500	Paired-end	23919636	150	Unpublished data. Private collection from CRAG
49	C5J7AACXX_2_27	Cucumis melo	Piel de Sapo	Flesh fruit-55 DAP	Illumina-HISeq 2500	Paired-end	31303445	150	Unpublished data. Private collection from CRAG
50	C5T43ACXX_7_23	Cucumis melo	Piel de Sapo	Flesh fruit-55 DAP	Illumina-HISeq 2500	Paired-end	51172145	150	Unpublished data. Private collection from CRAG
51	C5T4CACXX_4_25	Cucumis melo	Piel de Sapo	Flesh fruit-55 DAP	Illumina-HISeq 2500	Paired-end	42528511	150	Unpublished data. Private collection from CRAG
52	U489_RC_B00GGYU	Cucumis melo	Charentais-An	male early develop	Illumina-HISeq 2500	Paired-end	37238456	202	Unpublished data. Private collection from IPS2
53	U489_RC_B00GGYV	Cucumis melo	Charentais-An	male late develop	Illumina-HISeq 2500	Paired-end	42367890	202	Unpublished data. Private collection from IPS2

54	U489_RC_B00GGYW	Cucumis melo	Charentais-Gy	female early	Illumina-HISeq 2500	Paired-end	50387212	202	Unpublished data. Private collection from IPS2
55	U489_RC_B00GGYX	Cucumis melo	Charentais-Gy	female late	Illumina-HISeq 2500	Paired-end	37041156	202	Unpublished data. Private collection from IPS2
56	U489_RC_B00GGYY	Cucumis melo	Charentais-Her	hermaphrodite early	Illumina-HISeq 2500	Paired-end	41543607	202	Unpublished data. Private collection from IPS2
57	U489_RC_B00GGYZ	Cucumis melo	Charentais-Her	hermaphrodite late	Illumina-HISeq 2500	Paired-end	44189765	202	Unpublished data. Private collection from IPS2

Supplementary Table S4. List of masked genes. For each gene, the chromosome (chr), the start and end position, the Functional description (Annotation), the corresponding gene in the v3.5.1 release as well as the AED and score value of MAKER are reported.

Provided as Supplementary dataset.

Supplementary Table S5. The v4.0 annotation. For each annotated gene the transcript ID of the melon genome annotation v4.0, the position (chr, start and end), the orientation (strand), the functional description (Annotation), the AHRD score as well as the Gene Ontology annotation and the KEGG pathway association are reported. In addition, the correspondence of each gene with the previous melon genome annotation v3.5.1 (Garcia-Mas et al., 2012) is also reported.

Provided as Supplementary dataset.

Supplementary Table S6. Features comparison between Melon annotation v3.5.1 and v4.0. The increasing rate of v4.0 vs v3.5 is also presented.

**Feature**		**v3.5.1**	**v4.0**	**Increasing rate**
**Gene**	Number of genes	27.427	29.980	9,31%
	Mean gene length	2.776	3.577	28,85%
	Total genic length	76.125.905	107.251.060	40,89%
	Gene length range	150 ... 86,198	64...90,137	-57% ... 4.5%
	Gene density (kb/gene)	13,69	13,9	1,53%
	Number of transcripts (including alternatives for v3.5)	34.848	29.980	-13,97%
	Transcripts per gene	1,3	1	-23,08%
**Exon**	Number of exons*	112.683	146.210	29,75%
	Exons per transcript	4,6	4,87	5,81%
	Multi-exonic transcripts (%)	71,4	71,9	0,70%
	Mean exon length	271	275	1,48%
	Exon length range	2 ... 6,054	2 ... 10,325	0% ... 70.5%
	Total exon length*	32.513.969	40.078.907	23,30%
**Intron**	Number of introns*	85.256	116.230	36,30%
	Introns per transcript	3,6	3,87	7,50%
	Mean intron length	506	578	14,30%
	Intron length range	21 ... 76,916	5 ... 87,816	-76.2% ... 14.2%
	Total intron length*	43.611.936	67.172.153	54,02%
**CDS**	Number of CDS (including alternatives for v3.5)	34.848	29.980	-13,97%
	CDS per transcript	5,5	4,61	-16,18%
	Mean CDS length	950	974	2,53%
	CDS length range	78 ... 14,289	21 ... 15,702	-96.1% ... 9.8%
	Total cds length*	25.644.467	29.062.385	13,20%

**UTR**	Number of genes with at leat an UTR (5' OR 3')	15.776	22.471	42,44%
	Number of genes with both UTRs (5' AND 3')	10.504	15.960	51,94%
	Number of genes with 5’ UTR	13.925	18.917	35,85%
	Mean 5' UTR length	208	229	10,10%
	Total 5' UTR length*	2.899.836	4.340.988	49,70%
	5' UTR range	2 … 2,368	1 … 6,211	-50% ... 162%
	Number of genes with 3’ UTR	12.355	19.514	57,94%
	Mean 3' UTR length	429	340	-20,75%
	Total 3' UTR length*	5.308.027	6.637.197	25,04%
	3' UTR range	2 … 3,749	1 … 9,850	-50% ... 162.7%

* = the calculation was performed considering the longest alternative transcript in v3.5

Supplementary Table S7. Enzyme selection for optical mapping. The optimal restriction enzyme for optical mapping for the v3.5.1 assembly was selected by OpGen after testing 13 different restriction enzymes and evaluating different parameters of the restriction profile, such as average fragment size (Avg frag size), percentage of usable sequence (Usable %), number of large fragments (Frags > 100kb) and the size of the larger fragment (Max frag size).

Enzyme	Usable % 5kb – 20kb	Usable % 6kb – 15kb	Usable % 6kb – 12kb	Avg frag size (kb)	Frags > 100kb	Max frag size (kb)
AflII	22.57	5.55	5.55	3.7	0	49.92
BamHI	95.78	85.73	62.12	10.28	2	123.25
KpnI	73.47	44.14	18.01	14.02	17	142.06
NcoI	85.27	56.63	55	6.49	0	73.6
NheI	74.47	44.27	21.87	14.33	8	133.42
SpeI	63.67	40.61	40.08	4.92	0	73.71
BglII	10.41	2.66	2.66	3.89	0	42.83
EcoRI	1.27	0.09	0.09	3.35	0	41.02
MluI	15.51	7.49	2.48	23.84	183	310.55
NdeI	1.76	0.09	0.09	3.26	0	38.84
PvuII	93.31	82.63	66.44	10.36	3	113.69
XbaI	14.3	2.48	2.48	3.98	0	49.81
XhoI	97.51	88.91	74.36	9.16	1	107.04

Supplementary Table S8. Program parameters used.

Program	Parameters
STAR (version 2.5)	--outSAMtype BAM SortedByCoordinate<br>--outFilterMultimapNmax 1000<br>--alignIntronMax 20000<br>--alignEndsType EndToEnd<br>--outFilterMatchNminOverLread 0.3<br>--outFilterScoreMinOverLread 0.3<br>--alignMatesGapMax 500<br>--seedSearchStartLmax 40<br>--outFilterIntronMotifs RemoveNoncanonicalUnannotated<br>--alignSJoverhangMin 10<br>--twopassMode Basic
TRINITY (version 2.3.2)	--genome_guided_max_intron 20000<br>--jaccard_clip<br>--SS_lib_type RF

CD-HIT-EST<br>(version 4.6.6)	-c 0.95<br>-g 1<br>-r 0
MAKER<br>(version 2)	est_forward=1<br>est2genome=1<br>split_hit=20000<br>min_intron=20<br>single_exon=1<br>single_length=149<br>correct_est_fusion=1
SNAP<br>(HMM model)	always_complete=1<br>split_hit=10000<br>min_intron=20<br>single_exon=1<br>single_length=149<br>correct_est_fusion=1
